# On the Inclusion Relations of Global Ultradifferentiable Classes Defined by Weight Matrices

**DOI:** 10.1007/s00009-024-02694-1

**Published:** 2024-07-08

**Authors:** Chiara Boiti, David Jornet, Alessandro Oliaro, Gerhard Schindl

**Affiliations:** 1 Dipartimento di Matematica e Informatica, Università di Ferrara, Via Machiavelli n. 30, 44121 Ferrara, Italy; 2 Instituto Universitario de Matemática Pura y Aplicada IUMPA, Universitat Politècnica de València, Camino de Vera, s/n, 46022 Valencia, Spain; 3 Dipartimento di Matematica, Università di Torino, Via Carlo Alberto n. 10, 10123 Torino, Italy; 4 Fakultät für Mathematik, Universität Wien, Oskar-Morgenstern-Platz n. 1, 1090 Wien, Austria

**Keywords:** Gelfand–Shilov classes, Weight sequences, Weight functions, Weight matrices, Sequence spaces, 46A04, 46A45, 26E10

## Abstract

We study and characterize the inclusion relations of global classes in the general weight matrix framework in terms of growth relations for the defining weight matrices. We consider the Roumieu and Beurling cases, and as a particular case, we also treat the classical weight function and weight sequence cases. Moreover, we construct a weight sequence which is oscillating around any weight sequence which satisfies some minimal conditions and, in particular, around the critical weight sequence $(p!)^{1/2}$, related with the non-triviality of the classes. Finally, we also obtain comparison results both on classes defined by weight functions that can be defined by weight sequences and conversely.

## Introduction

We deal with global classes of ultradifferentiable functions defined by weight matrices and study and characterize different inclusion relations between these classes. There are basically two ways to introduce the classes of ultradifferentiable functions, the point of view of Komatsu [14], based on previous ideas of Carleman, which pays attention to the growth of the derivatives on compact sets modulated by a sequence $(M_p)_p$ of positive numbers, or the point of view of Björck [2], based on ideas of Beurling [1], who used a weight function to estimate the growth of the Fourier transform of compactly supported functions. Braun, Meise, and Taylor [7] unified these points of view by introducing weight functions which allow the use of convex analysis techniques. In terms of their topological structure, the classes are of two types, of Beurling type, which are classes whose topology looks like the topology of the space of all smooth functions, and of Roumieu type, whose topology looks like that of the space of real-analytic functions.

More recently, Rainer and Schindl [18] introduced weight matrices to study when the spaces of ultradifferentiable functions are closed under composition treating at the same time the classes in the sense of Komatsu (estimates of the derivatives with a sequence) and in the sense of Braun, Meise and Taylor (estimates of the derivatives via a weight function). They also studied intersections and inclusion relations of the classes in the local sense (i.e. when the estimates are given over the compact sets of a given open set). Since then several papers using weight matrices have been published. We mention, for instance, [4, 9, 10, 13], and the references therein. However, the characterization of inclusion relations in global classes of ultradifferentiable functions, i.e. classes where the estimates on the derivatives are taken in the whole of $\mathbb {R}^d$, has not been investigated yet. In this paper (Sect. 4), we characterize the inclusion relations of global classes defined by weight matrices using the isomorphisms introduced in [4, Sect. 5]. Moreover, given a weight sequence we construct an oscillating weight sequence around the first one to have examples of the opposite situation of the inclusion relations. In particular, in Sect. 3, we construct an oscillating weight sequence around the sequence $(p!)^{1/2}$, which is very related to the non-triviality of the corresponding ultradifferentiable class (see Remark 3.4). We begin with some notation (Sect. 2) and continue in Sect. 4 with the weight function case and the more general weight matrix case. In Sect. 5, we compare the classes when defined by weight functions and sequences in the spirit of [6]. Finally, in Sect. 6, we give alternative proofs in the non-quasianalytic case for the inclusion relations, which allows to eliminate some assumptions on the weight matrices with respect to the results of Sect. 4.

## Notation

### Weight Sequences

We denote $\mathbb {N}_0:=\mathbb {N}\cup \{0\}$. A *weight sequence* $\textbf{M}=(M_p)_{p\in \mathbb {N}_0}$ is a sequence of positive real numbers. A weight sequence $\textbf{M}=(M_p)_p$ is called *normalized* if $M_1\ge M_0=1$ (without loss of generality). We say that $\textbf{M}$ satisfies the *logarithmic convexity* condition, i.e. assumption (*M*1) of [14], if

2.1 $$\begin{aligned} M_p^2\le M_{p-1}M_{p+1},\qquad p\in \mathbb {N}. \end{aligned}$$

This is equivalent to the fact that the sequence of quotients $\mu _p:=\frac{M_p}{M_{p-1}}$, $p\in \mathbb {N}$, is nondecreasing and we set $\mu _0:=1$. If $\textbf{M}$ is normalized and log-convex, then

2.2 $$\begin{aligned} \forall \;p,q\in \mathbb {N}_0:\;\;\;M_pM_q\le M_{p+q}; \end{aligned}$$

see e.g. [20, Lemma 2.0.6]. Moreover, in this case, $\textbf{M}$ is nondecreasing because $\mu _p\ge \mu _1\ge 1$ for all $p\in \mathbb {N}$.

We say that $\textbf{M}$ satisfies *derivation closedness*, i.e. condition $(M2)'$ of [14], if

2.3 $$\begin{aligned} \exists \;D\ge 1\;\;\;M_{p+1}\le D^{p+1}M_p,\qquad p\in \mathbb {N}_0, \end{aligned}$$

and $\textbf{M}$ satisfies the stronger condition of *moderate growth*, i.e. condition (*M*2) of [14], if

2.4 $$\begin{aligned} \exists \;C\ge 1\;\;\;M_{p+q}\le C^{p+q}M_pM_q,\qquad p,q\in \mathbb {N}_0. \end{aligned}$$

For convenience, we set

$$\begin{aligned} {\mathcal{L}\mathcal{C}}:=\left\{ \textbf{M}\in \mathbb {R}_{>0}^{\mathbb {N}_0}:\;\textbf{M}\;\text {is normalized, log-convex},\;\lim _{p\rightarrow \infty }(M_p)^{1/p}=\infty \right\} , \end{aligned}$$

where $\mathbb {R}_{>0}^{\mathbb {N}_0}$ denotes the set of strictly positive sequences indexed in $\mathbb {N}_0$.

For a normalized sequence, $\textbf{M}=(M_p)_p$ the *associated function* is denoted by

2.5 $$\begin{aligned} \omega _{\textbf{M}}(t)=\sup _{p\in \mathbb {N}_0}\log \frac{|t|^p}{M_p},\qquad t\in \mathbb {R}, \end{aligned}$$

with the convention that $0^0:=1$ and $\log 0:=-\infty$. Note that condition $(M_p)^{1/p}\rightarrow +\infty$ is equivalent to $\omega _{\textbf{M}}(t)<+\infty$ by [4, Remark 1].

If $\textbf{M}\in {\mathcal{L}\mathcal{C}}$, then we can compute $\textbf{M}$ by involving $\omega _{\textbf{M}}$ as follows, see [16, Chapitre I, 1.4, 1.8] (and also [14, Proposition 3.2]):

2.6 $$\begin{aligned} M_p=\sup _{t\ge 0}\frac{t^p}{\exp (\omega _{\textbf{M}}(t))},\;\;\;p\in \mathbb {N}_0. \end{aligned}$$

Given two (normalized) sequences $\textbf{M}$ and $\textbf{N}$ we write $\textbf{M}\preceq \textbf{N}$, if

$$\begin{aligned} \exists \;C\ge 1\;\forall \;p\in \mathbb {N}_0:\;\;\;M_p\le C^pN_p. \end{aligned}$$

If $\textbf{M}\preceq \textbf{N}$ and $\textbf{N}\preceq \textbf{M}$, then we write $\textbf{M}\approx \textbf{N}$ and say that the sequences $\textbf{M}$ and $\textbf{N}$ are equivalent. Moreover, we write $\textbf{M}\vartriangleleft \textbf{N}$ if

$$\begin{aligned} \forall \;h>0\;\exists \;C_h\ge 1\;\forall \;p\in \mathbb {N}_0:\;\;\;M_p\le C_hh^pN_p\Longleftrightarrow \lim _{p\rightarrow \infty }\left( \frac{M_p}{N_p}\right) ^{1/p}=0. \end{aligned}$$

Next we recall [14, Lemmas 3.8 and 3.10] transferring these growth relations to the associated functions: Given $\textbf{M},\textbf{N}\in {\mathcal{L}\mathcal{C}}$, we have

$$\begin{aligned} \textbf{M}\preceq \textbf{N}\Longleftrightarrow \exists \;A,B\ge 1\;\forall \;t\ge 0:\;\,\;\omega _{\textbf{N}}(t)\le \omega _{\textbf{M}}(At)+B, \end{aligned}$$

and

$$\begin{aligned} \textbf{M}\vartriangleleft \textbf{N}\Longleftrightarrow \forall \;A>0\;\exists \;B\ge 1\;\forall \;t\ge 0:\;\,\;\omega _{\textbf{N}}(t)\le \omega _{\textbf{M}}(At)+B. \end{aligned}$$

The implications $\Rightarrow$ are clear by definition, for the converse one uses the fact that the sequences are log-convex and [14, (3.2)].

Similar conditions can be considered for sequences $\textbf{M}=(M_\alpha )_{\alpha \in \mathbb {N}_0^d}$ of positive real numbers for multi-indices $\alpha \in \mathbb {N}_0^d$ (see [4] for more details), i.e. for *multi-sequences*. In particular, normalization is given by $M_0=1$, $(M2)'$ is given by $M_{\alpha +e_i}\le A^{|\alpha |+1} M_\alpha$ for some $A\ge 1$, for any $\alpha \in \mathbb {N}_0^d$ and any $1\le i\le d$, $\textbf{M}\preceq \textbf{N}$ is given by $M_{\alpha }\le C^{|\alpha |}N_{\alpha }$ for some $C\ge 1$ and all $\alpha \in \mathbb {N}_0^d$ and similarly $\textbf{M}\vartriangleleft \textbf{N}$ by $\lim _{|\alpha |\rightarrow \infty }\left( \frac{M_{\alpha }}{N_{\alpha }}\right) ^{1/|\alpha |}=0$. However, the extension of the notion of logarithmic convexity for dimension $d>1$ in the anisotropic case is delicate, and we refer to [5] for more details. Obviously, in the isotropic case, this means that the weight sequence $\textbf{N}=(N_p)_{p\in \mathbb {N}_0}$ given by $N_p=M_{|\alpha |}=M_\alpha$ for $p=|\alpha |$ is logarithmically convex.

In the following, by abuse of notation, if $\textbf{M}$ is an isotropic weight multi-sequence, we will identify it (when needed) with the weight sequence $\textbf{N}$ defined as above.

### General Weight Matrices

Next we recall from [4, Sect. 3] the notion of weight matrices and global ultradifferentiable functions in the weight matrix setting. Let

2.7 $$\begin{aligned} \begin{aligned} \qquad \mathcal {M}:=\Big \{({\textbf {M}}^{(\lambda )})_{\lambda >0}:\ {}&{\textbf {M}}^{(\lambda )}= (M^{(\lambda )}_\alpha )_{\alpha \in \mathbb {N}_0^d},\ M^{(\lambda )}_0=1, \\ {}&{\textbf {M}}^{(\lambda )}\le {\textbf {M}}^{(\kappa )}\,\text { for } \text { all }\,0<\lambda \le \kappa \Big \}, \end{aligned} \end{aligned}$$

where $\textbf{M}^{(\lambda )}\le \textbf{M}^{(\kappa )}$ means that ${M}^{(\lambda )}_\alpha \le {M}^{(\kappa )}_\alpha$ for all $\alpha \in {\mathbb {N}}_0^d$. We call $\mathcal {M}$ a *weight matrix* and we say that it is *constant* if $\textbf{M}^{(\lambda )}\approx \textbf{M}^{(\kappa )}$ for all $\lambda ,\kappa >0$. In the one-dimensional case, we call $\mathcal {M}$ *standard log-convex* if $\textbf{M}^{(\lambda )}\in {\mathcal{L}\mathcal{C}}$ for any $\lambda >0$.

Given two weight matrices $\mathcal {M}=\{(\textbf{M}^{(\lambda )})_{\lambda >0}\}$ and $\mathcal {N}=\{(\textbf{N}^{(\lambda )})_{\lambda >0}\}$ we define the following three relevant growth conditions based on the weight sequence notation.

We write

$$\begin{aligned} \mathcal {M}(\preceq )\mathcal {N}{} & {} \qquad \quad \text{ if }\qquad \forall \;\lambda>0\;\exists \;\kappa>0:\;\;\;\textbf{M}^{(\kappa )}\preceq \textbf{N}^{(\lambda )},\\ \mathcal {M}\{\preceq \}\mathcal {N}{} & {} \qquad \quad \text{ if }\qquad \forall \;\lambda>0\;\exists \;\kappa>0:\;\;\;\textbf{M}^{(\lambda )}\preceq \textbf{N}^{(\kappa )},\\ \mathcal {M}\vartriangleleft \mathcal {N}{} & {} \qquad \quad \text{ if }\qquad \forall \;\lambda>0\;\forall \;\kappa >0:\;\;\;\textbf{M}^{(\lambda )}\vartriangleleft \textbf{N}^{(\kappa )}. \end{aligned}$$

We denote by $\Vert \cdot \Vert _\infty$ the supremum norm. Given a normalized weight sequence $\textbf{M}$, we consider the following spaces of weighted rapidly decreasing global ultradifferentiable functions of Roumieu type

$$\begin{aligned} \mathcal {S}_{\{{\textbf {M}}\}}(\mathbb {R}^d)&:=\Bigg \{f\in C^{\infty }(\mathbb {R}^d):\ \exists h>0\ \ \text { such } \text { that }\\ {}&\qquad \qquad \Vert f\Vert _{\infty ,{\textbf {M}},h}:=\sup _{\alpha ,\beta \in \mathbb {N}_0^d} \frac{\Vert x^\alpha \partial ^\beta f\Vert _\infty }{h^{|\alpha +\beta |}M_{\alpha +\beta }} <+\infty \Bigg \}, \end{aligned}$$

and of Beurling type

$$\begin{aligned} \mathcal {S}_{(\textbf{M})}(\mathbb {R}^d)&:=\{f\in C^\infty (\mathbb {R}^d):\ \forall h>0 \ \ \text{ we } \text{ have }\ \ \Vert f\Vert _{\infty ,\textbf{M},h}<+\infty \}, \end{aligned}$$

endowed with the inductive limit topology in the Roumieu case (which may be thought countable if we take $h\in \mathbb {N}$) and with the projective limit topology in the Beurling case (countable for $h^{-1}\in \mathbb {N}$). Next, the matrix type spaces are defined as follows:

$$\begin{aligned} \mathcal {S}_{\{\mathcal {M}\}}(\mathbb {R}^d)&:= \bigcup _{\lambda>0}\mathcal {S}_{\{\textbf{M}^{(\lambda )}\}} =\{f\in C^\infty (\mathbb {R}^d):\ \exists h,\lambda >0\ \\ {}&\text{ such } \text{ that }\ \ \Vert f\Vert _{\infty ,\textbf{M}^{(\lambda )},h}<+\infty \} \end{aligned}$$

in the Roumieu setting, and

$$\begin{aligned} \mathcal {S}_{(\mathcal {M})}(\mathbb {R}^d)&:= \bigcap _{\lambda>0}\mathcal {S}_{(\textbf{M}^{(\lambda )})}\\&=\{f\in C^\infty (\mathbb {R}^d):\ \forall h,\lambda >0\ \ \text{ we } \text{ have }\ \ \Vert f\Vert _{\infty ,\textbf{M}^{(\lambda )},h}<+\infty \} \end{aligned}$$

in the Beurling setting, again endowed with the inductive limit topology in the Roumieu case (which may be thought countable if we take $\lambda ,h\in \mathbb {N}$) and endowed with the projective limit topology in the Beurling case (countable for $\lambda ^{-1},h^{-1}\in \mathbb {N}$).

We denote by $\mathcal {E}_{\{\textbf{M}\}}, \mathcal {E}_{(\textbf{M})}, \mathcal {E}_{\{\mathcal {M}\}}, \mathcal {E}_{(\mathcal {M})}$ the analogous (local) classes of ultradifferentiable functions replacing $\Vert x^\alpha \partial ^\beta f\Vert _\infty$ by the supremum of $|\partial ^\beta f|$ on compact sets (and then take the projective limit over compact sets). Moreover, $\mathcal {D}_{\{\textbf{M}\}}, \mathcal {D}_{(\textbf{M})}, \mathcal {D}_{\{\mathcal {M}\}}, \mathcal {D}_{(\mathcal {M})}$ denote the corresponding classes of ultradifferentiable functions with compact support. We refer to [6] and [18] for precise definitions of such classes.

We collect here some conditions, already introduced in [4] and motivated by the assumptions in [15]. In the Roumieu case, we consider

2.8 $$\begin{aligned}{} & {} \begin{aligned}&\forall \lambda>0\ \exists \;\kappa \ge \lambda , B,C,H>0\ \forall \alpha ,\beta \in \mathbb {N}_0^d: \\&\alpha ^{\alpha /2}M^{(\lambda )}_\beta \le BC^{|\alpha |}H^{|\alpha +\beta |} M^{(\kappa )}_{\alpha +\beta }, \end{aligned} \end{aligned}$$

2.9 $$\begin{aligned}{} & {} \forall \;\lambda >0\;\exists \;\kappa \ge \lambda , A\ge 1\ \forall \alpha ,\beta \in \mathbb {N}_0^d:\ \ M^{(\lambda )}_{\alpha } M^{(\lambda )}_{\beta }\le A^{|\alpha +\beta |}M^{(\kappa )}_{\alpha +\beta }, \end{aligned}$$

2.10 $$\begin{aligned}{} & {} \forall \lambda >0\;\exists \kappa \ge \lambda ,A\ge 1\forall \alpha \in \mathbb {N}_0^d,1\le j\le d:\ M^{(\lambda )}_{\alpha +e_j}\le A^{|\alpha |+1}M^{(\kappa )}_\alpha ,\nonumber \\ \end{aligned}$$

2.11 $$\begin{aligned}{} & {} \forall \lambda >0\exists \kappa \ge \lambda ,A\ge 1\forall \alpha ,\beta \in \mathbb {N}_0^d:\ M^{(\lambda )}_{\alpha +\beta }\le A^{|\alpha +\beta |} M^{(\kappa )}_{\alpha } M^{(\kappa )}_{\beta }, \end{aligned}$$

and in the Beurling case

2.12 $$\begin{aligned}{} & {} \begin{aligned}&\forall \;\lambda>0\ \exists \;0<\kappa \le \lambda , H>0\ \forall C>0\ \exists B>0\ \forall \alpha ,\beta \in \mathbb {N}_0^d:\\&\alpha ^{\alpha /2}M^{(\kappa )}_\beta \le BC^{|\alpha |}H^{|\alpha +\beta |} M^{(\lambda )}_{\alpha +\beta }, \end{aligned} \end{aligned}$$

2.13 $$\begin{aligned}{} & {} \forall \;\lambda >0\;\exists \;0<\kappa \le \lambda , A\ge 1\ \forall \alpha ,\beta \in \mathbb {N}_0^d:\ \ M^{(\kappa )}_{\alpha } M^{(\kappa )}_{\beta }\le A^{|\alpha +\beta |}M^{(\lambda )}_{\alpha +\beta }, \end{aligned}$$

2.14 $$\begin{aligned}{} & {} \forall \lambda >0\;\exists 0<\kappa \le \lambda ,A\ge 1\forall \alpha \in \mathbb {N}_0^d,1\le j\le d:\ M^{(\kappa )}_{\alpha +e_j}\le A^{|\alpha |+1}M^{(\lambda )}_\alpha , \end{aligned}$$

2.15 $$\begin{aligned}{} & {} \forall \lambda >0\exists 0<\kappa \le \lambda ,A\ge 1\forall \alpha ,\beta \in \mathbb {N}_0^d:\ M^{(\kappa )}_{\alpha +\beta }\le A^{|\alpha +\beta |}M^{(\lambda )}_{\alpha } M^{(\lambda )}_{\beta }. \end{aligned}$$

We summarize now some consequences for a given weight matrix $\mathcal {M}$ as defined in (2.7):

(i) By [4, Proposition 1] the spaces $\mathcal {S}_{\{\mathcal {M}\}}(\mathbb {R}^d)$ resp. $\mathcal {S}_{(\mathcal {M})}(\mathbb {R}^d)$ can be equivalently defined in terms of the system of (weighted) $L^2$-seminorms when assuming (2.8) and (2.10) in the Roumieu case, resp. (2.12) and (2.14) in the Beurling case.
(ii) If $\mathcal {M}$ satisfies (2.9) and (2.11), then we can replace in the definition of $\mathcal {S}_{\{\mathcal {M}\}}(\mathbb {R}^d)$ the seminorm $\Vert \cdot \Vert _{\infty ,\textbf{M}^{(\lambda )},h}$ by
  2.16 $$\begin{aligned} \sup _{\alpha ,\beta \in \mathbb {N}_0^d} \frac{\Vert x^\alpha \partial ^\beta f\Vert _\infty }{h^{|\alpha +\beta |}M^{(\lambda )}_{\alpha }M^{(\lambda )}_{\beta }}. \end{aligned}$$
  We have an analogous statement for the class $\mathcal {S}_{(\mathcal {M})}(\mathbb {R}^d)$ when assuming (2.13) and (2.15). When we define the spaces $\mathcal {S}_{\{\mathcal {M}\}}(\mathbb {R}^d)$ or $\mathcal {S}_{(\mathcal {M})}(\mathbb {R}^d)$ with the weighted $L^2$ norms, the similar property holds.

### Weight Functions

#### Definition 2.1

A *weight function* is a continuous increasing function $\omega \!:[0,+\infty )\rightarrow [0,+\infty )$ such that

$$(\alpha )$$
$$\exists L\ge 1\ \forall t\ge 0:\ \omega (2t)\le L(\omega (t)+1)$$;
$$(\beta )$$
$$\omega (t)=O(t^2)$$ as $$t\rightarrow +\infty $$;
$$(\gamma )$$
$$\log t=o(\omega (t))$$ as $$t\rightarrow +\infty $$;
$$(\delta )$$
$\varphi _\omega (t):=\omega (e^t)$ is convex on $[0,+\infty )$.

Then we define $\omega (t):=\omega (|t|)$ if $t\in \mathbb {R}^d$, where |*t*| denotes the Euclidean norm of *t*.

We call $\omega$ a *general weight function,* if $\omega$ satisfies all listed properties except $(\beta )$.

It is not restrictive to assume $\omega |_{[0,1]}\equiv 0$ (normalization). As usual, we define the Young conjugate $\varphi ^*_{\omega }$ of $\varphi _{\omega }$ by

$$\begin{aligned} \varphi ^*_{\omega }(s):=\sup _{t\ge 0}\{ts-\varphi _{\omega }(t)\},\;\;\;s\ge 0, \end{aligned}$$

which is an increasing convex function such that $$\varphi ^{**}_{\omega }=\varphi _{\omega }$$ and $$s\mapsto \frac{\varphi ^{*}_{\omega }(s)}{s}$$ is increasing. Condition $$(\gamma )$$ guarantees that $$\varphi ^*_{\omega }$$ is finite.

We introduce the following growth relations between two (general) weight functions arising naturally in the ultradifferentiable framework: We write

$$\begin{aligned} \omega \preceq \sigma \quad \text{ if }\quad \sigma (t)=O(\omega (t)),\;\;\;t\rightarrow \infty , \end{aligned}$$

and

$$\begin{aligned} \omega \vartriangleleft \sigma \quad \text{ if }\quad \sigma (t)=o(\omega (t)),\;\;\;t\rightarrow \infty . \end{aligned}$$

If $\omega \preceq \sigma$ and $\sigma \preceq \omega$ are valid, then we write $\omega \sim \sigma$ and call the weights *equivalent.*

For any given (general) weight function $\omega$, we set

2.17 $$\begin{aligned} W^{(\lambda )}_\alpha :=e^{\frac{1}{\lambda }\varphi ^*_{\omega }(\lambda |\alpha |)}, \qquad \lambda >0,\alpha \in \mathbb {N}_0^d, \end{aligned}$$

and consider the weight matrix

2.18 $$\begin{aligned} \mathcal {M}_{\omega }:=(\textbf{W}^{(\lambda )})_{\lambda>0}=(W^{(\lambda )}_\alpha )_{\lambda >0,\,\alpha \in \mathbb {N}_0^d}. \end{aligned}$$

We observe that growth relations between weight functions and their corresponding associated weight matrices are connected as follows:

#### Lemma 2.2

Let $\omega$ and $\sigma$ be general weight functions. Then the following are equivalent:

- $$\mathcal {M}_{\omega }(\preceq )\mathcal {M}_\sigma $$,
- $$\mathcal {M}_{\omega }\{\preceq \}\mathcal {M}_\sigma $$,
- $$\omega \preceq \sigma $$.

Moreover, the following are equivalent:

(d) $$\mathcal {M}_{\omega }\vartriangleleft \mathcal {M}_\sigma $$,
(e) $$\omega \vartriangleleft \sigma $$.

#### Proof

The equivalences (a)–(c) are explained in statement $(1')$ inside the proof of [18, Corollary 5.17].

The equivalence between (*d*) and (*e*) follows from [18, Proposition (2), Theorem 5.14 (2), Corollary 5.17 (2)]. $\square$

We recall now the following result, which was proved in [4, Lemma 11] for a weight function, but which can be stated for a *general weight function*, since assumption $(\beta )$ was not needed in the proof.

#### Lemma 2.3

Let $\omega$ be a (general) weight function. Then $\mathcal {M}_{\omega }$ satisfies the following properties:

(i) $$W^{(\lambda )}_0=1,\quad \lambda >0$$;
(ii) $(W^{(\lambda )}_\alpha )^2\le W^{(\lambda )}_{\alpha -e_i}W^{(\lambda )}_{\alpha +e_i},\quad \lambda >0,\alpha \in \mathbb {N}^d_0$ with $\alpha _i\ne 0$, and $i=1,\dots ,d$;
(iii) $$\textbf{W}^{(\kappa )}\le \textbf{W}^{(\lambda )},\quad 0<\kappa \le \lambda $$;
(iv) $$W^{(\lambda )}_{\alpha +\beta }\le W^{(2\lambda )}_\alpha W^{(2\lambda )}_\beta ,\quad \lambda >0,\alpha ,\beta \in \mathbb {N}_0^d$$;
(v) $$\forall h>0\ \exists A\ge 1\ \forall \lambda >0\ \exists D\ge 1\ \forall \alpha \in \mathbb {N}_0^d:\ \ \ h^{|\alpha |}W^{(\lambda )}_\alpha \le DW^{(A\lambda )}_\alpha ;$$
(vi) Both conditions (2.10) and (2.14) are valid.
(vii) Conditions (2.9) and (2.13) are satisfied for $\kappa =\lambda$ and $A=1$.

The spaces of rapidly decreasing $\omega$-ultradifferentiable functions are then defined as follows: In the Roumieu case,

$$\begin{aligned} \mathcal {S}_{\{\omega \}}(\mathbb {R}^d)&:=\Bigg \{f\in C^{\infty }(\mathbb {R}^d):\ \exists \lambda>0\ \text{ s.t. }\sup _{\alpha ,\beta \in \mathbb {N}_0^d}\Vert x^{\alpha }\partial ^{\beta } f\Vert _{\infty } e^{-\frac{1}{\lambda }\varphi ^*_\omega (\lambda |\alpha +\beta |)}<+\infty \Bigg \}\\&=\Bigg \{f\in C^\infty (\mathbb {R}^d):\ \exists \lambda >0\ \text{ s.t. }\ \Vert f\Vert _{\infty ,\textbf{W}^{(\lambda )}}:= \sup _{\alpha ,\beta \in \mathbb {N}_0^d}\frac{\Vert x^\alpha \partial ^\beta f\Vert _\infty }{W^{(\lambda )}_{\alpha +\beta }}<+\infty \Bigg \}, \end{aligned}$$

and in the Beurling case,

$$\begin{aligned} \mathcal {S}_{(\omega )}(\mathbb {R}^d):=\big \{f\in C^{\infty }(\mathbb {R}^d):\ \forall \lambda >0,\ \Vert f\Vert _{\infty ,\textbf{W}^{(\lambda )}}<+\infty \big \}. \end{aligned}$$

From (*iv*), (*vii*) in Lemma 2.3, we have that equivalently the classes can be treated by separated growth at infinity, i.e.

$$\begin{aligned} \mathcal {S}_{\{\omega \}}(\mathbb {R}^d)=\Bigg \{f\in C^{\infty }(\mathbb {R}^d):\ \exists \lambda >0\ \ \text{ such } \text{ that }\ \sup _{\alpha ,\beta \in \mathbb {N}_0^d} \frac{\Vert x^{\alpha }\partial ^{\beta }f\Vert _\infty }{W^{(\lambda )}_{\alpha } W^{(\lambda )}_{\beta }}<+\infty \Bigg \} \end{aligned}$$

and

$$\begin{aligned} \mathcal {S}_{(\omega )}(\mathbb {R}^d)=\Bigg \{f\in C^{\infty }(\mathbb {R}^d):\ \forall \lambda >0,\ \sup _{\alpha ,\beta \in \mathbb {N}_0^d}\frac{\Vert x^{\alpha }\partial ^{\beta }f\Vert _\infty }{W^{(\lambda )}_{\alpha } W^{(\lambda )}_{\beta }}<+\infty \Bigg \}. \end{aligned}$$

We can also insert $h^{|\alpha +\beta |}$ at the denominator (for some $h>0$ in the Roumieu case and for all $h>0$ in the Beurling case) by (*v*) in Lemma 2.3. In particular, we finally recall from [4, Proposition 5] (where again assumption $(\beta )$ was not necessary) that, analogously as in the ultradifferentiable setting, we can use the associated weight matrix in order to have an alternative and useful description of the classes defined by weight matrices:

#### Proposition 2.4

Let $\omega$ be a (general) weight function and $\mathcal {M}_{\omega }$ be the weight matrix defined in (2.17), (2.18). We have

$$\begin{aligned} \mathcal {S}_{\{\mathcal {M}_{\omega }\}}(\mathbb {R}^d)=\mathcal {S}_{\{\omega \}}(\mathbb {R}^d)\hspace{20pt}\text {and}\hspace{20pt} \mathcal {S}_{(\mathcal {M}_{\omega })}(\mathbb {R}^d)=\mathcal {S}_{(\omega )}(\mathbb {R}^d), \end{aligned}$$

and both equalities are also topological.

We refer to [3] for a more complete characterization of such spaces, and to [7] for the analogous spaces $\mathcal {E}_{\{\omega \}}/\mathcal {D}_{\{\omega \}}$ and $\mathcal {E}_{(\omega )}/\mathcal {D}_{(\omega )}$ of ultradifferentiable functions/with compact support.

## Oscillating Sequences and a Critical Example Case

The aim of this section is to construct explicitly a weight sequence $\textbf{M}$ which oscillates around a given fixed sequence $\textbf{N}\in {\mathcal{L}\mathcal{C}}$. We assume for $\textbf{N}$ some more basic growth properties and show that these requirements can be transferred to $\textbf{M}$, too. Moreover, these conditions yield the fact that the function $\omega \equiv \omega _{\textbf{M}}$ is also oscillating around the weight $\omega _{\textbf{N}}$. Since by construction $\textbf{M}\in {\mathcal{L}\mathcal{C}}$, we focus on the one-dimensional situation (or, equivalently, on the isotropic case, i.e. $M_{\alpha }:=M_{|\alpha |}$). As a special case, we apply this to $\textbf{N}\equiv \textbf{G}^{1/2}:=(p!^{1/2})_{p\in {\mathbb {N}}_0}$ and the corresponding weight function $\omega (t)=t^2$ in the sense that $\omega \equiv \omega _{\textbf{G}^{1/2}}$ (see Example 3.1). This is a crucial case since it is related to the problem of non-triviality of $\mathcal {S}_{(\omega )}$ and $\mathcal {S}_{\{\omega \}}$ (see Remark 3.4).

We construct the sequence $\textbf{M}$ in terms of the quotients $\mu _p:=\frac{M_p}{M_{p-1}}$ by putting $M_p:=\prod _{i=1}^p\mu _i$ (and $M_0:=1$, empty product) and consider the associated weight function $\omega _{\textbf{M}}$. More precisely, the aim is to show that

(i) $$\omega _{\textbf{M}}$$ satisfies $$(\alpha ), (\gamma ), (\delta )$$,
(ii) $$\inf _{p\ge 1}\left( \frac{M_p}{N_p}\right) ^{1/p}=0$$ and
(iii) $$\sup _{p\ge 1}\left( \frac{M_p}{N_p}\right) ^{1/p}=+\infty $$.

Concerning (*i*), first, we recall that for any given $\textbf{M}\in {\mathcal{L}\mathcal{C}}$ the function $\omega _{\textbf{M}}$ satisfies automatically the basic assumption in Definition 2.1 and conditions $(\gamma )$ and $(\delta )$, see [16, Chapitre I], [14, Definition 3.1] and [6, Lemma 12 $(4)\Rightarrow (5)$].

We start the construction as follows. Let from now on $(\alpha _j)_{j\ge 0}$ be a sequence of positive real numbers such that

3.1 $$\begin{aligned} 1<\alpha _{\min }:=\inf _{j\ge 0}\alpha _j\le \sup _{j\ge 0}\alpha _j=:\alpha _{\max }<+\infty . \end{aligned}$$

Moreover let $Q\in \mathbb {N}$, $Q\ge 2$, be given, arbitrary but fixed. We introduce a new sequence $(\beta _j)_{j\ge 1}$ by

3.2 $$\begin{aligned} \beta _1=\cdots =\beta _{Q-1}:=\alpha _0^{\frac{1}{Q-1}} \end{aligned}$$

and

3.3 $$\begin{aligned} \beta _{Q^n}=\cdots =\beta _{Q^{n+1}-1}:=\alpha _n^{\frac{1}{Q^n(Q-1)}},\;\;\;n\in \mathbb {N}. \end{aligned}$$

Finally, $\textbf{M}$ is defined via the quotients $(\mu _j)_{j\ge 1}$ as follows: We put

3.4 $$\begin{aligned} \mu _1:=c\ge 1,\hspace{30pt}\mu _{j+1}:=\beta _j\mu _j,\;\;\;j\ge 1. \end{aligned}$$

Using this, we have $\frac{\mu _{Qj}}{\mu _j}=\frac{\mu _{j+1}}{\mu _j}\cdots \frac{\mu _{Qj}}{\mu _{Qj-1}}=\beta _j\cdots \beta _{Qj-1}$ for all $j\ge 1$ which yields a product consisting of $j(Q-1)$-many factors.

*Claim I:* $\alpha _{\min }\le \frac{\mu _{Qj}}{\mu _j}\le \alpha _{\max }$ is valid for all $j\in \mathbb {N}$.

If $$j=1$$, then $$\frac{\mu _{Qj}}{\mu _j}=\frac{\mu _Q}{\mu _1}=\beta _1\cdots \beta _{Q-1}=\alpha _0^{\frac{Q-1}{Q-1}}=\alpha _0$$.

If $j=Q^n$, $n\in \mathbb {N}$ arbitrary, then $Qj=Q^{n+1}$ and so $\frac{\mu _{Qj}}{\mu _j}{=}\beta _{Q^n}\cdots \beta _{Q^{n+1}-1}=\alpha _n^{\frac{Q^{n+1}-Q^n}{Q^n(Q-1)}}=\alpha _n$.

If $Q^n<j\le Q^{n+1}-1$, $n\in \mathbb {N}$ arbitrary, then $Q^{n+1}<Qj\le Q^{n+2}-Q<Q^{n+2}-1$ and we get, for $i=Q^{n+1}-j$,

$$\begin{aligned} \frac{\mu _{Qj}}{\mu _j}&=\beta _j\cdots \beta _{Qj-1}=\alpha _n^{\frac{i}{Q^n(Q-1)}}\cdot \alpha _{n+1}^{\frac{j(Q-1)-i}{Q^{n+1}(Q-1)}}\ge \alpha _{\min }^{\frac{Qi+Qj-j-i}{Q^{n+1}(Q-1)}}\\&=\alpha _{\min }^{\frac{(i+j)(Q-1)}{Q^{n+1}(Q-1)}}=\alpha _{\min }^{\frac{(i+j)}{Q^{n+1}}}=\alpha _{\min }. \end{aligned}$$

Finally, if $Q^0=1<j\le Q-1$ (when $Q\ge 3$), then we can estimate as before replacing $\alpha _n$ and $\alpha _{n+1}$ by $\alpha _0$ and $\alpha _1$ respectively. The estimate from above in the claim is obtained analogously when taking $\alpha _{\max }$ instead of $\alpha _{\min }$.

*Claim II:* $\textbf{M}\in {\mathcal{L}\mathcal{C}}$ holds.

Since $\alpha _{\min }>1$, we clearly have that $\beta _j>1$ for all $j\in \mathbb {N}$, which is equivalent to $\mu _{j+1}>\mu _j$ for all $j\in \mathbb {N}$. Hence, $\textbf{M}$ is log-convex. The previous Claim I yields $\mu _{Qj}\ge \alpha _{\min }\mu _j$ for all $j\in \mathbb {N}$, thus by iteration $\mu _{Q^nj}\ge \alpha _{\min }^n\mu _j$ for all $n\in \mathbb {N}$. Consequently, we get $\mu _{Q^n}\ge \alpha _{\min }^n\mu _1=\alpha _{\min }^nc\ge \alpha _{\min }^n$ which tends to infinity as $n\rightarrow \infty$ because $\alpha _{\min }>1$ by assumption. This proves $\lim _{j\rightarrow \infty }\mu _j=+\infty$, hence $\lim _{j\rightarrow \infty }(M_j)^{1/j}=+\infty$ follows (e.g. see [18, p. 104]).

Now we start with the definition of $\textbf{M}$ in terms of the aforementioned construction using the auxiliary sequences $(\beta _j)_j$ resp. $(\alpha _j)_j$. We put

$$\begin{aligned} \mu _0=\mu _1:=1(=c), \end{aligned}$$

so $M_1=M_0=1$ follows which ensures normalization. The idea is now to define $\textbf{M}$ (via $(\alpha _j)_j$) piece-wise by considering an increasing sequence (of integers) $(k_j)_{j\ge 1}$. Given a sequence $\textbf{N}\in {\mathcal{L}\mathcal{C}}$, we consider the sequence of its quotients

$$\begin{aligned} \nu _k=\frac{N_k}{N_{k-1}}, \quad k=1,2,\ldots , \end{aligned}$$

and, moreover, we assume that $\textbf{N}$ satisfies

3.5 $$\begin{aligned} \exists \;Q\in \mathbb {N}:\;\;\;1<\liminf _j\frac{\nu _{Qj}}{\nu _j}\ \ \text {and }\ \ \sup _j\frac{\nu _{2j}}{\nu _j}<+\infty . \end{aligned}$$

It is immediate that $Q\ge 2$ in the above requirement and (3.5) is crucial to ensure that $\omega _{\textbf{M}}$ satisfies $(\alpha )$ (see (*III*) below) and that $\textbf{M}$ has moderate growth (see (*IV*) below).

Let now *Q* be the parameter according to (3.5) and without loss of generality $Q\ge 3$. First, we set $k_1:=Q$ and

$$\begin{aligned} \alpha _0=\alpha _1:=4\sqrt{\nu _{k_1}}. \end{aligned}$$

Then we have

$$\begin{aligned} \mu _{k_1}=\mu _Q:=\alpha _0\mu _1=\alpha _0=4\sqrt{\nu _{k_1}}(\ge 4), \end{aligned}$$

and for $1<i<k_1$, we have put $\mu _i:=\beta _{i-1}\mu _{i-1}=\alpha _0^{\frac{1}{Q-1}}\mu _{i-1}$ (see (3.2)).

In the next step, we select a number $n_1\in \mathbb {N}$, $n_1\ge 2$, and put $k_2:=Q^{n_1}k_1=Q^{n_1+1}>k_1$. Here we choose $n_1$ sufficiently large in order to ensure $\nu _{Q^{n_1}k_1}>64\nu _{k_1}$ (note that $\lim _{j\rightarrow \infty }\nu _j=\infty$). Then we set

$$\begin{aligned} \alpha _2=\cdots =\alpha _{n_1}:=64^{-\frac{1}{n_1-1}}\left( \frac{\nu _{k_2}}{\nu _{k_1}}\right) ^{\frac{1}{n_1-1}}>1, \end{aligned}$$

and get $\alpha _0\cdot \alpha _1\cdot \alpha _2\cdots \alpha _{n_1}=\displaystyle 16\nu _{k_1}\frac{1}{64}\frac{\nu _{k_2}}{\nu _{k_1}}=\frac{1}{4}\nu _{k_2}$. Hence, by (3.3) and (3.4) we get

$$\begin{aligned} \frac{\mu _{k_2}}{\mu _{k_1}}=\frac{\mu _{Q^{n_1+1}}}{\mu _Q}=\prod ^{Q^{n_1+1}-1}_{i=Q}\frac{\mu _{i+1}}{\mu _i}=\prod _{l=1}^{n_1}\prod _{i=Q^l}^{Q^{l+1}-1}\beta _i=\alpha _1\cdots \alpha _{n_1}, \end{aligned}$$

and so one has

$$\begin{aligned} \mu _{k_2}=\mu _{Q^{n_1}k_1}=\mu _{k_1}\cdot \alpha _1\cdots \alpha _{n_1}=\alpha _0\cdot \alpha _1\cdot \alpha _2\cdots \alpha _{n_1}=\frac{1}{4}\nu _{k_2}. \end{aligned}$$

Note that for $k_1<i<k_2$ we put $\mu _i:=\alpha _{l+1}^{\frac{1}{Q^{l+1}(Q-1)}}\mu _{i-1}$ whenever $Q^lk_1<i\le Q^{l+1}k_1$, $0\le l\le n_1-1$ [see (3.3) and (3.4)].

Then select a number $n_2\in \mathbb {N}$, $n_2\ge 2$, put $k_3:=Q^{n_2}k_2=Q^{n_2+n_1+1}>k_2$ and

$$\begin{aligned} \alpha _{n_1+1}=\cdots =\alpha _{n_1+n_2}:=32^{\frac{1}{n_2}}\left( \frac{\nu _{k_3}}{\nu _{k_2}}\right) ^{\frac{1}{n_2}}(\ge 32). \end{aligned}$$

Hence, we get

$$\begin{aligned} \alpha _0\cdot \alpha _1\cdot \alpha _2\cdots \alpha _{n_1+n_2}=\frac{1}{4}\nu _{k_2}32\frac{\nu _{k_3}}{\nu _{k_2}}=8\nu _{k_3}. \end{aligned}$$

So

$$\begin{aligned} \mu _{k_3}=\mu _{Q^{n_2}k_2}=\alpha _0\cdot \alpha _1\cdot \alpha _2\cdots \alpha _{n_1+n_2}=8\nu _{k_3}, \end{aligned}$$

since $$\frac{\mu _{k_3}}{\mu _{k_2}}=\prod ^{Q^{n_2+n_1+1}-1}_{i=Q^{n_1+1}}\frac{\mu _{i+1}}{\mu _i}=\alpha _{n_1+1}\cdots \alpha _{n_1+n_2}$$.

For $k_2<i<k_3$, again according to (3.3) and (3.4), we have put $\mu _i:=\alpha _{l+n_1+1}^{\frac{1}{Q^{l+n_1+1}(Q-1)}}\mu _{i-1}$ whenever $Q^lk_2<i\le Q^{l+1}k_2$, $0\le l\le n_2-1$.

And then we proceed as follows:

*Case I—from odd to even numbers.* Given any $k_j$ with $j\ge 3$ odd, then we select $n_j\in \mathbb {N}$, $n_j\ge 2$, put $k_{j+1}:=Q^{n_j}k_j=Q^{n_j+\cdots +n_1+1}$ and define

$$\begin{aligned} \alpha _{n_{j-1}+\cdots + n_1+1}=\cdots =\alpha _{n_j+n_{j-1}+\cdots +n_1}:=(2^j(j+1))^{-\frac{1}{n_j}}\left( \frac{\nu _{k_{j+1}}}{\nu _{k_{j}}}\right) ^{\frac{1}{n_j}}. \end{aligned}$$

*Case II—from even to odd numbers.* Given any $k_j$ with $j\ge 4$ even, then we select $n_j\in \mathbb {N}$, $n_j\ge 2$, put $k_{j+1}:=Q^{n_j}k_j=Q^{n_j+\cdots +n_1+1}$ and define

$$\begin{aligned} \alpha _{n_{j-1}+\cdots + n_1+1}=\cdots =\alpha _{n_j+n_{j-1}+\cdots +n_1}:=(2^{j+1}j)^{\frac{1}{n_j}}\big (\frac{\nu _{k_{j+1}}}{\nu _{k_{j}}}\big )^{\frac{1}{n_j}}. \end{aligned}$$

With these choices, first, for all odd $j\ge 3$ (starting with the case $j=3$ from above), one has

$$\begin{aligned}&\alpha _0\alpha _1\alpha _2\cdots \alpha _{n_j+n_{j-1}+\cdots +n_1}=\left( \alpha _0\alpha _1\alpha _2\cdots \alpha _{n_{j-1}+\cdots + n_1}\right) \cdot \\&\alpha _{n_{j-1}+\cdots + n_1+1}\cdots \alpha _{n_j+\cdots +n_1} =2^{j}\nu _{k_j}\frac{1}{2^j(j+1)}\cdot \frac{\nu _{k_{j+1}}}{\nu _{k_{j}}}=\frac{1}{j+1}\nu _{k_{j+1}}, \end{aligned}$$

and so

$$\begin{aligned}&\mu _{k_{j+1}}=\mu _{Q^{n_j}k_j}=\alpha _0\alpha _1\alpha _2\cdots \alpha _{n_j+\cdots +n_1}=\frac{1}{j+1}\nu _{k_{j+1}}. \end{aligned}$$

On the other hand, for all even $j\ge 4$, we see

$$\begin{aligned}&\alpha _0\alpha _1\alpha _2\cdots \alpha _{n_j+n_{j-1}+\cdots +n_1}=\left( \alpha _0\alpha _1\alpha _2\cdots \alpha _{n_{j-1}+\cdots + n_1}\right) \cdot \\&\alpha _{n_{j-1}+\cdots + n_1+1}\cdots \alpha _{n_j+\cdots +n_1} =\frac{1}{j}\nu _{k_j}\cdot 2^{j+1}j \frac{\nu _{k_{j+1}}}{\nu _{k_{j}}}=2^{j+1}\nu _{k_{j+1}}, \end{aligned}$$

and so

$$\begin{aligned}&\mu _{k_{j+1}}=\mu _{Q^{n_j}k_j}=\alpha _0\alpha _1\alpha _2\cdots \alpha _{n_j+\cdots +n_1}=2^{j+1}\nu _{k_{j+1}}. \end{aligned}$$

Moreover, recall that  and for all $k_j<i<k_{j+1}$, according to (3.3) and (3.4), we have set*Claim III:* (3.5) implies (3.1). First, we treat the upper estimates and note that for Case I, we have $(2^j(j+1))^{-\frac{1}{n_j}}\big (\frac{\nu _{k_{j+1}}}{\nu _{k_{j}}}\big )^{\frac{1}{n_j}}\le \big (\frac{\nu _{k_{j+1}}}{\nu _{k_{j}}}\big )^{\frac{1}{n_j}}\le A$ for some $A\ge 1$. The second estimate is equivalent to $\frac{\nu _{Q^{n_j}k_j}}{\nu _{k_{j}}}\le A^{n_j}$ (recall: $k_{j+1}=Q^{n_j}k_j$) and this is valid because by, the second part of (3.5), we have $\frac{\nu _{2j}}{\nu _j}\le B$ for some $B\ge 1$ and all $j\in \mathbb {N}_0$ and so, iterating this estimate $cn_j$-times with $c\in \mathbb {N}$ such that $Q\le 2^c$, we have $\frac{\nu _{Q^{n_j}k_j}}{\nu _{k_j}}\le \frac{\nu _{2^{cn_j}k_j}}{\nu _{k_j}}\le B^{cn_j}=A^{n_j}$ with $A:=B^c$. Here the first estimate holds by the log-convexity of $\textbf{N}$.

For Case II with the expression $(2^{j+1}j)^{\frac{1}{n_j}}\big (\frac{\nu _{k_{j+1}}}{\nu _{k_{j}}}\big )^{\frac{1}{n_j}}$, we get the bound $(2^{j+1}j)^{\frac{1}{n_j}}A$ by the previous comments. And this can be bounded uniformly for all even $j\ge 4$ by some $A_1>A$ when choosing $n_j$ large enough. Therefore, note that *A* is not depending on the choice of $n_j$; it only depends on given (fixed) constants *Q* and *B*, both depending only on $\textbf{N}$ via (3.5). Summarizing, the upper estimate in (3.1) is verified for all $j\in \mathbb {N}$ since the remaining cases are only finitely many indices.

Now we treat the lower estimate. By the first part in (3.5), we have that there exists some $\epsilon >0$ such that (by iteration) $\frac{\nu _{k_{j+1}}}{\nu _{k_j}}=\frac{\nu _{Q^{n_j}k_j}}{\nu _{k_j}}\ge (1+\epsilon )^{n_j}$ provided that $k_j$ is chosen sufficiently large. We assume now that we have chosen $k_3$ large enough (for a fixed $\epsilon >0$) and so the above estimate holds for all $j\ge 3$. Now, concerning Case I for all odd $j\ge 3$ we estimate by $(2^j(j+1))^{-\frac{1}{n_j}}\big (\frac{\nu _{k_{j+1}}}{\nu _{k_{j}}}\big )^{\frac{1}{n_j}}\ge (2^j(j+1))^{-\frac{1}{n_j}}(1+\epsilon )>1$ and the last estimate is equivalent to requiring $(1+\epsilon )^{n_j}>2^j(j+1)$. This can be achieved by choosing $n_j$, $j\ge 3$ odd, sufficiently large.

Concerning Case II, we observe $(2^{j+1}j)^{\frac{1}{n_j}}\big (\frac{\nu _{k_{j+1}}}{\nu _{k_{j}}}\big )^{\frac{1}{n_j}}\ge \big (\frac{\nu _{k_{j+1}}}{\nu _{k_{j}}}\big )^{\frac{1}{n_j}}\ge (1+\epsilon )$ for all even $j\ge 4$.

To guarantee $\alpha _j>1$ for all $j\in \mathbb {N}$, i.e. also for $1\le j\le n_1+n_2$, we recall our choice for $n_1$ above.

Summarizing, we get:

(I) $\textbf{M}\in {\mathcal{L}\mathcal{C}}$: Normalization is obtained as seen above, log-convexity holds by the fact that $\alpha _j>1$ for all $j\in \mathbb {N}$ and so the sequence $j\mapsto \mu _j$ is (strictly) increasing. Since for all odd $j\ge 3$ by construction we get $\mu _{k_j}\ge 2^{j+1}\nu _{k_j}$, we see that $\lim _{j\rightarrow \infty }\mu _j=+\infty$ (since $\nu _{k_j}$ is nondecreasing by the logarithmic convexity), and so also $\lim _{j\rightarrow \infty }(M_j)^{1/j}=+\infty$, e.g. see [18, p. 104].
(II) Moreover, by Claim III, we see that $1<\alpha _{\min }\le \alpha _{\max }<+\infty$ when choosing $n_j$ large enough. Thus, by construction and Claim I, we have $1<\liminf _{j\rightarrow \infty }\frac{\mu _{Qj}}{\mu _j}\le \limsup _{j\rightarrow \infty }\frac{\mu _{Qj}}{\mu _j}<+\infty$.
(III) The proof of [6, Lemma 12, $(2)\Rightarrow (4)$] shows that the lower estimate $1<\liminf _{j\rightarrow \infty }\frac{\mu _{Qj}}{\mu _j}$ implies $(\alpha )$ for $\omega _{\textbf{M}}$. Thus, $\omega _{\textbf{M}}$ has all standard requirements to be a weight function except $(\beta )$, i.e. $\omega _{\textbf{M}}$ is a general weight function.
(IV) By the upper estimate, $\textbf{M}$ satisfies (2.4) (see e.g. [19, Lemma 2.2]). Equivalently, by taking into account [14, Proposition 3.6], the associated weight function $\omega _{\textbf{M}}$ satisfies the following condition
  3.6 $$\begin{aligned} \exists \;H\ge 1\;\forall \;t\ge 0:\;\;\;2\omega (t)\le \omega (Ht)+H, \end{aligned}$$
  introduced in [6, Corollary 16(3)] in order to compare ultradifferentiable spaces defined by weight sequences $(M_p)_p$ and weight functions $\omega _{\textbf{M}}$.
(V) Let now $\mathcal {M}_{\omega _{\textbf{M}}}=\{\textbf{M}^{(\lambda )}: \lambda >0\}$ be the matrix associated to $\omega _{\textbf{M}}$. By [18, Lemma 5.9] and (3.6) it follows that $\textbf{M}^{(\lambda )}\approx \textbf{M}^{(\kappa )}$, i.e. $\mathcal {M}_{\omega _{\textbf{M}}}$ is constant. In this case, we get $\textbf{M}\equiv \textbf{M}^{(1)}$ by definition of $\textbf{M}^{(1)}$ and [14, Proposition 3.2] (see also the proof of [21, Theorem 6.4]):
  $$\begin{aligned} M^{(1)}_p&:=\exp (\varphi ^{*}_{\omega _{{\textbf {M}}}}(p))=\exp \left( \sup _{y\ge 0}\{py-\omega _{{\textbf {M}}}(e^y)\}\right) =\sup _{y\ge 0}\exp (py-\omega _{{\textbf {M}}}(e^y))\\ {}&=\sup _{y\ge 0}\frac{\exp (py)}{\exp (\omega _{{\textbf {M}}}(e^y))}=\sup _{t\ge 1}\frac{t^p}{\exp (\omega _{{\textbf {M}}}(t))}=\sup _{t\ge 0}\frac{t^p}{\exp (\omega _{{\textbf {M}}}(t))}=M_p. \end{aligned}$$
  Note that by normalization, we have $\omega _{\textbf{M}}(t)=0$ for $0\le t\le 1$ which follows by the known integral representation formula for $\omega _M$, see [16, 1.8. III] and also [14, (3.11)], and since $t^p\le 1$ for $0\le t\le 1$, $p\in \mathbb {N}_0$ arbitrary. Consequently, $\textbf{M}^{(\lambda )}\approx \textbf{M}$ for all $\lambda >0$ and so
  $$\begin{aligned}{} & {} \mathcal {S}_{\{\omega _{\textbf{M}}\}}({\mathbb {R}}^d)=\mathcal {S}_{\{\mathcal {M}_{\omega _{\textbf{M}}}\}}({\mathbb {R}}^d)=\mathcal {S}_{\{\textbf{M}\}}({\mathbb {R}}^d),\\ {}{} & {} \mathcal {S}_{(\omega _{\textbf{M}})}({\mathbb {R}}^d)=\mathcal {S}_{(\mathcal {M}_{\omega _{\textbf{M}}})}({\mathbb {R}}^d)=\mathcal {S}_{(\textbf{M})}({\mathbb {R}}^d), \end{aligned}$$
  as topological vector spaces.
(VI) By construction, we have $\mu _{k_j}=2^j\nu _{k_j}$ for all $j\ge 3$ odd and $\mu _{k_j}=\frac{1}{j}\nu _{k_j}$ for all $j\ge 4$ even. Thus,
  $$\begin{aligned} \liminf _{p\rightarrow \infty }\frac{\mu _p}{\nu _p}=0\quad \text{ and } \quad \limsup _{p\rightarrow \infty }\frac{\mu _p}{\nu _p}=+\infty . \end{aligned}$$
  Now,
  $$\begin{aligned} \exists \;A\ge 1\;\forall \;p\in \mathbb {N}:\;\;\;(M_p)^{1/p}\le \mu _p\le A(M_p)^{1/p}. \end{aligned}$$
  In fact, the first estimate follows by log-convexity and normalization (see e.g. [20, Lemma 2.0.4]), the second one by moderate growth, e.g. see again [19, Lemma 2.2]. Consequently the sequences $(M_p^{1/p})_p$ and $(\mu _p)_p$ are comparable up to a constant. By (3.5), the same is valid for $\textbf{N}$ and so we have
  $$\begin{aligned} \liminf _{p\rightarrow \infty }\left( \frac{M_p}{N_p}\right) ^{1/p}=0,\hspace{30pt}\limsup _{p\rightarrow \infty }\left( \frac{M_p}{N_p}\right) ^{1/p}=+\infty . \end{aligned}$$
  Hence, $\textbf{M}$ and $\textbf{N}$ are not comparable, which means that neither $\textbf{M}\preceq \textbf{N}$ nor $\textbf{N}\preceq \textbf{M}$ hold (consequently, neither $\textbf{M}\vartriangleleft \textbf{N}$ nor $\textbf{N}\vartriangleleft \textbf{M}$, too).

### Example 3.1

Now we treat the case when $\textbf{N}$ is the critical sequence $\textbf{G}^{1/2}:=(p!^{1/2})_{p\in \mathbb {N}_0}\in {\mathcal{L}\mathcal{C}}$. It is not difficult to see that $\textbf{N}$ fulfills the requirements to find a weight sequence $\textbf{M}_0$ such that the weight function $\omega _0\equiv \omega _{\textbf{M}_0}$ oscillates around the critical weight function $\omega (t)=t^2$. This is related to the problem of non-triviality of the classes $\mathcal {S}_{(\omega )}({\mathbb {R}}^d)$ and $\mathcal {S}_{\{\omega \}}({\mathbb {R}}^d)$ as we explain below in Remark 3.4.

Now, translating into the notation of growth relations of [4, Lemma 13] (whose proof did not use assumption $(\beta )$), we obtain:

### Lemma 3.2

Let $\omega$ be a (general) weight function. Then

$$\begin{aligned} t\mapsto t^2\preceq \omega \Longleftrightarrow \omega (t)=O(t^2)\Longleftrightarrow \;\forall \;\lambda >0:\;\textbf{G}^{1/2}\preceq \textbf{W}^{(\lambda )}, \end{aligned}$$

and

$$\begin{aligned} t\mapsto t^2\vartriangleleft \omega \Longleftrightarrow \omega (t)=o(t^2)\Longleftrightarrow \;\forall \;\lambda >0:\;\textbf{G}^{1/2}\vartriangleleft \textbf{W}^{(\lambda )}. \end{aligned}$$

Similarly, following the lines in the proof of [4, Lemma 13], we obtain:

### Lemma 3.3

Let $\omega$ be a (general) weight function. Then

$$\begin{aligned} \omega \preceq t\mapsto t^2\Longleftrightarrow t^2=O(\omega (t))\Longleftrightarrow \;\forall \;\lambda >0:\;\textbf{W}^{(\lambda )}\preceq \textbf{G}^{1/2}, \end{aligned}$$

and

$$\begin{aligned} \omega \vartriangleleft t\mapsto t^2\Longleftrightarrow t^2=o(\omega (t))\Longleftrightarrow \;\forall \;\lambda >0:\;\textbf{W}^{(\lambda )}\vartriangleleft \textbf{G}^{1/2}. \end{aligned}$$

Note that these results follow also from [18, Lemma 5.16, Corollary 5.17] and we can replace in all conditions “$\forall \;\lambda >0$” equivalently by “$\exists \;\lambda >0$”.

Now, for $\textbf{M}_0\equiv \textbf{W}^{(\lambda )}_0$, which is equivalent to the weight matrix associated to the general weight function $\omega _0$ of Example 3.1, we have that $\textbf{M}_0$ and $\textbf{G}^{1/2}$ are not comparable. This means that neither $\textbf{M}_0\preceq \textbf{G}^{1/2}$ nor $\textbf{G}^{1/2}\preceq \textbf{M}_0$ hold (hence neither $\textbf{M}_0\vartriangleleft \textbf{G}^{1/2}$ nor $\textbf{G}^{1/2}\vartriangleleft \textbf{M}_0$ hold, too). It also follows from Lemmas 3.2 and 3.3 that neither $\omega _0\preceq t\mapsto t^2$ nor $t\mapsto t^2\preceq \omega _0$ are valid (and hence neither $\omega _0\vartriangleleft t\mapsto t^2$ nor $t\mapsto t^2\vartriangleleft \omega _0$, too).

Finally, we mention that $\textbf{M}_0$ does not satisfy the requirements of [6] because their basic assumption

$$\begin{aligned} \exists c > 0: \quad (c(p + 1))^p\le M_p, \qquad p\in {\mathbb {N}}_0, \end{aligned}$$

is violated, since

3.7 $$\begin{aligned} \liminf _{p\rightarrow \infty }\left( \frac{M_p}{p!^{1/2}}\right) ^{1/p}=0, \end{aligned}$$

and then also $\liminf _{p\rightarrow \infty }\left( \frac{M_p}{p!}\right) ^{1/p}=0$.

Moreover, from (3.7) again, we also get that the sequence $\textbf{M}_0$ cannot satisfy the conditions in [4, Proposition 3]. Hence, the spaces $\mathcal {S}_{(\textbf{M}_0)}({\mathbb {R}}^d)$ and $\mathcal {S}_{\{\textbf{M}_0\}}({\mathbb {R}}^d)$ do not contain any Hermite function. Still, we do not know if these classes are non-trivial. However, the existence of such an oscillating sequence is important in view of the following:

### Remark 3.4

Let $\omega$ be a given (general) weight function according to Definition 2.1. If $\omega (t)=o(t^2)$, then [4, Corollary 3(b)] yields that $\mathcal {S}_{(\omega )}({\mathbb {R}}^d)$ is non-trivial (all Hermite functions are contained in this class).

However, when $t^2=O(\omega (t))$ as $t\rightarrow \infty$, then we prove now that $\mathcal {S}_{(\omega )}({\mathbb {R}}^d)=\{0\}$: First, for any $f\in \mathcal {S}_{(\omega )}({\mathbb {R}}^d)$, we get

$$\begin{aligned} \forall \;\lambda >0:\;\;\;\sup _{x\in \mathbb {R}^d}|f(x)|e^{\lambda \omega (x)}<\infty ,\hspace{30pt}\sup _{\xi \in \mathbb {R}^d}|\widehat{f}(\xi )|e^{\lambda \omega (\xi )}<\infty , \end{aligned}$$

which gives, by the relation $t^2=O(\omega (t))$,

$$\begin{aligned} \sup _{x\in \mathbb {R}^d}|f(x)|e^{|x|^2/2}<\infty ,\hspace{30pt}\sup _{\xi \in \mathbb {R}^d}|\widehat{f}(\xi )|e^{|\xi |^2/2}<\infty . \end{aligned}$$

Now [11, Corollary] yields $f\equiv 0$.

Analogously, in the Roumieu case, $\omega (t)=O(t^2)$ implies by [4, Corollary 3(a)] that $\mathcal {S}_{\{\omega \}}({\mathbb {R}}^d)$ is non-trivial but $t^2=o(\omega (t))$ implies $\mathcal {S}_{\{\omega \}}({\mathbb {R}}^d)=\{0\}$.

## Characterization of the Inclusion Relations of Global Ultradifferentiable Classes

In this section, we characterize the inclusion relations of spaces of rapidly decreasing ultradifferentiable functions using the isomorphisms with sequence spaces obtained in [4], inspired by the previous ideas by Langenbruch [15]. Such isomorphisms are obtained assigning to each function its coefficients in its Hermite expansion; see also [24]. Let us distinguish the various cases.

### The Weight Matrix Case

In this case, for a weight matrix $\mathcal {M}$, we recall the isomorphisms proved in [4, Theorem 1]. In the Roumieu case, if conditions (2.8) and (2.10) are satisfied, then

4.1 $$\begin{aligned} \mathcal {S}_{\{\mathcal {M}\}}(\mathbb {R}^d)\cong \Lambda _{\{\mathcal {M}\}}:=\Bigg \{{} & {} \!\!\textbf{c}=(c_{\alpha })_{\alpha \in \mathbb {N}^d_0}\in \mathbb {C}^{\mathbb {N}_0^d}:\nonumber \\{} & {} \exists l\in \mathbb {N}:\ \Vert \textbf{c}\Vert _{\textbf{M}^{(l)},l}:=\sup _{\alpha \in \mathbb {N}^d_0}|c_{\alpha }|e^{\omega _{\textbf{M}^{(l)}}(\frac{\sqrt{\alpha }}{l})}<+\infty \Bigg \}.\nonumber \\ \end{aligned}$$

Analogously in the Beurling case, if conditions (2.12) and (2.14) are satisfied, then

4.2 $$\begin{aligned} \mathcal {S}_{(\mathcal {M})}(\mathbb {R}^d)\cong \Lambda _{(\mathcal {M})}:=\Bigg \{{} & {} \!\!\textbf{c}=(c_{\alpha })_{\alpha \in \mathbb {N}^d_0}\in \mathbb {C}^{\mathbb {N}_0^d}:\nonumber \\{} & {} \forall l\in \mathbb {N}:\ \Vert \textbf{c}\Vert _{\textbf{M}^{(1/l)},\frac{1}{l}}:=\sup _{\alpha \in \mathbb {N}^d_0}|c_{\alpha }|e^{\omega _{\textbf{M}^{(1/l)}}(\sqrt{\alpha }l)}<+\infty \Bigg \}.\nonumber \\ \end{aligned}$$

We start with the Roumieu case.

#### Theorem 4.1

Let $\mathcal {M}:=\{\textbf{M}^{(\lambda )}: \lambda >0\}$ and $\mathcal {N}:=\{\textbf{N}^{(\lambda )}: \lambda >0\}$ be given weight matrices and consider the following assertions:

(i) $$\mathcal {M}\{\preceq \}\mathcal {N}$$,
(ii) $\mathcal {S}_{\{\mathcal {M}\}}(\mathbb {R}^d)\subseteq \mathcal {S}_{\{\mathcal {N}\}}(\mathbb {R}^d)$ holds with continuous inclusion.

Then we get the following: $(i)\Rightarrow (ii)$ is valid for all dimensions $d\in \mathbb {N}$. If (*ii*) holds for the case $d=1$ and both matrices are standard log-convex with (2.8) and (2.10), then $(ii)\Rightarrow (i)$ is valid, too.

#### Proof

The implication $(i)\Rightarrow (ii)$ follows by the definition of the spaces.

For $(ii)\Rightarrow (i)$, we use the inclusion for the dimension $d=1$ and so the matrices consist only of sequences $\textbf{M}^{(\lambda )},\textbf{N}^{(\lambda )}\in {\mathcal{L}\mathcal{C}}$.

By the assumptions on $\mathcal {M}$ and $\mathcal {N}$, we can apply the isomorphism (4.1) and so (*ii*) yields $\Lambda _{\{\mathcal {M}\}}\cong \mathcal {S}_{\{\mathcal {M}\}}(\mathbb {R})\subseteq \mathcal {S}_{\{\mathcal {N}\}}(\mathbb {R})\cong \Lambda _{\{\mathcal {N}\}}$.

We consider the sequence $\textbf{c}:=(c_k)_{k\in \mathbb {N}_0}\in \mathbb {C}^{\mathbb {N}_0}$ defined by $c_k:=e^{-\omega _{\textbf{M}^{(j)}}(\frac{\sqrt{k}}{j})}$ with $j\in \mathbb {N}$, $j\ge 2$, arbitrary but from now on fixed. So $\textbf{c}\in \Lambda _{\{\mathcal {M}\}}$ follows by choosing $l=j$ in (4.1) and this yields $\textbf{c}\in \Lambda _{\{\mathcal {N}\}}$ as well. Thus

$$\begin{aligned} \forall \;j\in \mathbb {N}\;\exists \;l\in \mathbb {N}\;\exists \;C\ge 1\;\forall \;k\in \mathbb {N}_0:\;\;\;e^{-\omega _{\textbf{M}^{(j)}}(\frac{\sqrt{k}}{j})}=|c_k|\le Ce^{-\omega _{\textbf{N}^{(l)}}(\frac{\sqrt{k}}{l})}, \end{aligned}$$

which implies $$\log (C)+\omega _{\textbf{M}^{(j)}}(\sqrt{k})\ge \log (C)+\omega _{\textbf{M}^{(j)}}(\frac{\sqrt{k}}{j})\ge \omega _{\textbf{N}^{(l)}}(\frac{\sqrt{k}}{l})$$.

Let now $t\in \mathbb {R}$ with $\sqrt{k}<t<\sqrt{k+1}$ for some $k\in \mathbb {N}$. Then

$$\begin{aligned} \omega _{\textbf{N}^{(l)}}\left( \frac{t}{l}\right)&\le \omega _{\textbf{N}^{(l)}} \left( \frac{\sqrt{k+1}}{l}\right) \le \log (C)+\omega _{\textbf{M}^{(j)}} \left( \frac{\sqrt{k+1}}{j}\right) \le \log (C)+\omega _{\textbf{M}^{(j)}}(\sqrt{k})\\&\le \log (C)+\omega _{\textbf{M}^{(j)}}(t). \end{aligned}$$

Here we have used that $\frac{\sqrt{k+1}}{j}\le \sqrt{k}$ is valid for any $k\in \mathbb {N}$ when $j\ge 2$ and that each $\omega _{\textbf{M}^{(j)}}$ is increasing. Finally, if $0<t<1$, then $\omega _{\textbf{N}^{(l)}}\left( \frac{t}{l}\right) \le \omega _{\textbf{N}^{(l)}}\left( \frac{1}{l}\right)$. Consequently, by enlarging the constant *C* if necessary, so far we have shown

$$\begin{aligned} \forall \;j\in \mathbb {N},\;j\ge 2,\;\exists \;l\in \mathbb {N}\;\exists \;C\ge 1\;\forall \;t\ge 0:\;\;\;\omega _{\textbf{N}^{(l)}}\left( \frac{t}{l}\right) \le \log (C)+\omega _{\textbf{M}^{(j)}}(t). \end{aligned}$$

We use this estimate and the fact that each sequence belonging to the matrices is log-convex and normalized. Hence, by (2.6), we get for all $p\in \mathbb {N}_0$:

$$\begin{aligned} M^{(j)}_p&=\sup _{t\ge 0}\frac{t^p}{\exp (\omega _{\textbf{M}^{(j)}}(t))}\le C\sup _{t\ge 0}\frac{t^p}{\exp (\omega _{\textbf{N}^{(l)}}(\frac{t}{l}))}=C\sup _{s\ge 0}\frac{(sl)^p}{\exp (\omega _{\textbf{N}^{(l)}}(s))}=Cl^pN^{(l)}_p, \end{aligned}$$

which proves

$$\begin{aligned} \forall \;j\in \mathbb {N},\;j\ge 2,\;\exists \;l\in \mathbb {N}:\;\;\;\textbf{M}^{(j)}\preceq \textbf{N}^{(l)} \end{aligned}$$

and so $\mathcal {M}\{\preceq \}\mathcal {N}$ is verified. Note that the assumption $j\ge 2$ is not restricting the generality in our considerations since we are dealing with Roumieu type spaces. $\square$

Next we treat the mixed situation between the Roumieu case and the Beurling case.

#### Theorem 4.2

Let $\mathcal {M}:=\{\textbf{M}^{(\lambda )}: \lambda >0\}$ and $\mathcal {N}:=\{\textbf{N}^{(\lambda )}: \lambda >0\}$ given weight matrices and consider the following assertions:

(i) $$\mathcal {M}\vartriangleleft \mathcal {N}$$,
(ii) $\mathcal {S}_{\{\mathcal {M}\}}(\mathbb {R}^d)\subseteq \mathcal {S}_{(\mathcal {N})}(\mathbb {R}^d)$ holds with continuous inclusion.

Then we get the following: $(i)\Rightarrow (ii)$ is valid, for all dimensions $d\in \mathbb {N}$. If (*ii*) holds for the case $d=1$ and if both matrices are standard log-convex and $\mathcal {M}$ does have (2.8) and (2.10), whereas $\mathcal {N}$ is required to satisfy (2.12) and (2.14), then $(ii)\Rightarrow (i)$ is valid, too.

#### Proof

Again, $(i)\Rightarrow (ii)$ follows by the definition of the spaces.

For $(ii)\Rightarrow (i)$, we use this inclusion for $d=1$. By the assumptions on $\mathcal {M}$ and $\mathcal {N}$ and the isomorphisms (4.1)–(4.2), we have that (*ii*) yields $\Lambda _{\{\mathcal {M}\}}\cong \mathcal {S}_{\{\mathcal {M}\}}(\mathbb {R})\subseteq \mathcal {S}_{(\mathcal {N})}(\mathbb {R})\cong \Lambda _{(\mathcal {N})}$. As in the previous proof, we consider the sequence $\textbf{c}:=(c_k)_{k\in \mathbb {N}_0}\in \mathbb {C}^{\mathbb {N}_0}$ defined by $c_k:=e^{-\omega _{\textbf{M}^{(j)}}(\frac{\sqrt{k}}{j})}$ with $j\in \mathbb {N}$, $j\ge 2$, arbitrary but from now on fixed. So $\textbf{c}\in \Lambda _{\{\mathcal {M}\}}$ by choosing $l=j$ and now the assumption yields $\textbf{c}\in \Lambda _{(\mathcal {N})}$ as well. Thus, we obtain

$$\begin{aligned}&{} \forall \;j\in \mathbb {N},\;j\ge 2,\;\forall \;l\in \mathbb {N}\;\exists \;C\ge 1\;\forall \;k\in \mathbb {N}_0:\;\;\;\\{}&{} \ \qquad \qquad \qquad e^{-\omega _{{\textbf {M}}^{(j)}}(\frac{\sqrt{k}}{j})} =|c_k|\le Ce^{-\omega _{{\textbf {N}}^{(1/l)}}(\sqrt{k}l)}, \end{aligned}$$

which gives $\log (C)+\omega _{\textbf{M}^{(j)}}(\sqrt{k})\ge \log (C)+\omega _{\textbf{M}^{(j)}}(\frac{\sqrt{k}}{j})\ge \omega _{\textbf{N}^{(1/l)}}(\sqrt{k}l)$ and note that the arising constant *C* is depending on *l* and *j*.

Let now $t\in \mathbb {R}$ with $\sqrt{k}<t<\sqrt{k+1}$ for some $k\in \mathbb {N}$. Then

$$\begin{aligned} \omega _{\textbf{N}^{(1/l)}}(tl)&\le \omega _{\textbf{N}^{(1/l)}}(\sqrt{k+1}l)\le \log (C)+\omega _{\textbf{M}^{(j)}}\left( \frac{\sqrt{k+1}}{j}\right) \le \log (C)+\omega _{\textbf{M}^{(j)}}(\sqrt{k})\\&\le \log (C)+\omega _{\textbf{M}^{(j)}}(t), \end{aligned}$$

as in the proof of Theorem 4.1. Finally, if $0<t<1$, then $\omega _{\textbf{N}^{(1/l)}}(tl)\le \omega _{\textbf{N}^{(1/l)}}(l)$. Consequently, by enlarging the constant *C* if necessary, so far we have shown

$$\begin{aligned} \forall \;j\in \mathbb {N}\;j\ge 2,\;\forall \;l\in \mathbb {N}\;\exists \;C\ge 1\;\forall \;t\ge 0:\;\;\;\omega _{\textbf{N}^{(1/l)}}(tl)\le \log (C)+\omega _{\textbf{M}^{(j)}}(t). \end{aligned}$$

We use this estimate and the fact that each sequence belonging to the matrices is log-convex and normalized, hence by (2.6), we get for all $p\in \mathbb {N}_0$ and $i\le l$:

$$\begin{aligned} M^{(j)}_p&=\sup _{t\ge 0}\frac{t^p}{\exp (\omega _{\textbf{M}^{(j)}}(t))}\le C\sup _{t\ge 0}\frac{t^p}{\exp (\omega _{\textbf{N}^{(1/l)}}(tl))}=C\sup _{s\ge 0}\frac{(s/l)^p}{\exp (\omega _{\textbf{N}^{(1/l)}}(s))} \\ {}&=C\frac{1}{l^p}N^{(1/l)}_p \le C\frac{1}{l^p}N^{(1/i)}_p. \end{aligned}$$

This estimate proves $\textbf{M}^{(j)}\vartriangleleft \textbf{N}^{(1/i)}$ for all $i,j\in \mathbb {N}$, $j\ge 2$: Let *i* and $j\ge 2$ be arbitrary but fixed, then we get by the previous computations that $\left( \frac{M^{(j)}_p}{N^{(1/i)}_p}\right) ^{1/p}\le C_l^{1/p}\frac{1}{l}$ for all $l\ge i$ and $p\in \mathbb {N}$. Assumption $j\ge 2$ is not restricting since the matrix $\mathcal {M}$ is related to Roumieu-type conditions and small indices can be omitted without changing the corresponding function class. Thus, we have verified $\mathcal {M}\vartriangleleft \mathcal {N}$. $\square$

Finally, we treat the general weight matrix case in the Beurling-type setting.

#### Theorem 4.3

Let $\mathcal {M}:=\{\textbf{M}^{(\lambda )}: \lambda >0\}$ and $\mathcal {N}:=\{\textbf{N}^{(\lambda )}: \lambda >0\}$ be given and consider the following assertions:

(i) $$\mathcal {M}(\preceq )\mathcal {N}$$,
(ii) $\mathcal {S}_{(\mathcal {M})}(\mathbb {R}^d)\subseteq \mathcal {S}_{(\mathcal {N})}(\mathbb {R}^d)$ holds with continuous inclusion.

Then we get the following: $(i)\Rightarrow (ii)$ is valid for all dimensions $d\in \mathbb {N}$. If (*ii*) holds for the case $d=1$, both matrices are standard log-convex with (2.12) and (2.14), then $(ii)\Rightarrow (i)$ is valid, too.

#### Proof

Again, $(i)\Rightarrow (ii)$ follows by the definition of the spaces.

$(ii)\Rightarrow (i)$ We use this inclusion for $d=1$. By the assumptions on $\mathcal {M}$ and $\mathcal {N}$ and the isomorphism (4.2), we have that (*ii*) yields $\Lambda _{(\mathcal {M})}\cong \mathcal {S}_{(\mathcal {M})}(\mathbb {R}^d)\subseteq \mathcal {S}_{(\mathcal {N})}(\mathbb {R}^d)\cong \Lambda _{(\mathcal {N})}$ with continuous inclusion. By the continuity of the inclusion, we get

4.3 $$\begin{aligned} \forall \;j\in \mathbb {N}\;\exists \;l\in \mathbb {N}\;\exists \;C\ge 1\;\forall \;\textbf{c}\in \Lambda _{(\mathcal {M})}:\;\;\;\Vert \textbf{c}\Vert _{\textbf{N}^{(1/j)},\frac{1}{j}}\le C\Vert \textbf{c}\Vert _{\textbf{M}^{(1/l)},\frac{1}{l}}. \end{aligned}$$

For $i\in \mathbb {N}_0$, we consider the sequence $\textbf{c}^i$ defined by $c^i_k:=\delta _{i,k}$. It is clear that each $\textbf{c}^i\in \Lambda _{(\mathcal {M})}$ because $\Vert \textbf{c}^i\Vert _{\textbf{M}^{(1/j)},\frac{1}{j}}=e^{\omega _{\textbf{M}^{(1/j)}}(\sqrt{i}j)}<+\infty$ for all $i\in \mathbb {N}_0$ and $j\in \mathbb {N}$. We apply (4.3) to the family $\textbf{c}^i$, $i\in \mathbb {N}_0$, and get

$$\begin{aligned} \forall \;j\in \mathbb {N}\;\exists \;l\in \mathbb {N}\;\exists \;C\ge 1\forall \;k\in \mathbb {N}_0:\;\;\;e^{\omega _{\textbf{N}^{(1/j)}}(\sqrt{k}j)}\le Ce^{\omega _{\textbf{M}^{(1/l)}}(\sqrt{k}l)}, \end{aligned}$$

consequently $\omega _{\textbf{N}^{(1/j)}}(\sqrt{k}j)\le \log (C)+\omega _{\textbf{M}^{(1/l)}}(\sqrt{k}l)\le \log (C)+\omega _{\textbf{M}^{(1/l)}}(\sqrt{k}2l)$ follows because each $\omega _{\textbf{M}^{(j)}}$ is increasing.

Let now $t\in \mathbb {R}$ with $\sqrt{k}<t<\sqrt{k+1}$ for some $k\in \mathbb {N}$. Then

$$\begin{aligned} \omega _{\textbf{N}^{(1/j)}}(tj)&\le \omega _{\textbf{N}^{(1/j)}}(\sqrt{k+1}j)\le \log (C) +\omega _{\textbf{M}^{(1/l)}}(\sqrt{k+1}l) \\ {}&\le \log (C)+\omega _{\textbf{M}^{(1/l)}}(\sqrt{k}2l)\\&\le \log (C)+\omega _{\textbf{M}^{(1/l)}}(t2l). \end{aligned}$$

Here we have used that $\sqrt{k+1}\le 2\sqrt{k}$.

If $t\in \mathbb {R}$ with $0<t<1$, then $\omega _{\textbf{N}^{(1/j)}}(tj)\le \omega _{\textbf{N}^{(1/j)}}(j)$. Consequently, by enlarging the constant *C* if necessary, so far we have shown

$$\begin{aligned} \forall \;j\in \mathbb {N}\;\exists \;l\in \mathbb {N}\;\exists \;C\ge 1\;\forall \;t\ge 0:\;\;\;\omega _{\textbf{N}^{(1/j)}}(t)\le \log (C)+\omega _{\textbf{M}^{(1/l)}}(t2l/j). \end{aligned}$$

Finally, using this and again (2.6), we get for all $p\in \mathbb {N}_0$:

$$\begin{aligned} N^{(1/j)}_p&=\sup _{t\ge 0}\frac{t^p}{\exp (\omega _{\textbf{N}^{(1/j)}}(t))}\ge \frac{1}{C}\sup _{t\ge 0}\frac{t^p}{\exp (\omega _{\textbf{M}^{(1/l)}}(t2l/j))}\\ {}&=\frac{1}{C}\sup _{s\ge 0}\frac{(sj/(2l))^p}{\exp (\omega _{\textbf{M}^{(1/l)}}(s))}\\&=\frac{1}{C}\left( \frac{j}{2l}\right) ^pM^{(1/l)}_p, \end{aligned}$$

which proves $\textbf{M}^{(1/l)}\preceq \textbf{N}^{(1/j)}$ and so $\mathcal {M}(\preceq )\mathcal {N}$ is verified. $\square$

### The Single Weight Sequence Case

It is straight-forward to obtain the analogous results for Theorems 4.1, 4.2 and 4.3 in the single weight sequence case and we get the following characterization:

#### Theorem 4.4

Let $\textbf{M},\textbf{N}$ be two weight normalized multi-sequences such that both satisfy the condition of derivation closedness $(M2)'$.

(I) Let $\textbf{M}$ and $\textbf{N}$ satisfy (2.8) and consider the following assertions:
  (i) $$\textbf{M}\preceq \textbf{N}$$,
  (ii) $\mathcal {S}_{\{\textbf{M}\}}(\mathbb {R}^d)\subseteq \mathcal {S}_{\{\textbf{N}\}}(\mathbb {R}^d)$ with continuous inclusion.
(II) Let $\textbf{M}$ and $\textbf{N}$ satisfy (2.8) and (2.12) respectively, and consider the following assertions:
  (i) $$\textbf{M}\vartriangleleft \textbf{N}$$,
  (ii) $\mathcal {S}_{\{\textbf{M}\}}(\mathbb {R}^d)\subseteq \mathcal {S}_{(\textbf{N})}(\mathbb {R}^d)$ with continuous inclusion.
(III) Let $\textbf{M}$ and $\textbf{N}$ satisfy (2.12) and consider the following assertions:
  (i) $$\textbf{M}\preceq \textbf{N}$$,
  (ii) $\mathcal {S}_{(\textbf{M})}(\mathbb {R}^d)\subseteq \mathcal {S}_{(\textbf{N})}(\mathbb {R}^d)$ with continuous inclusion.

Then all implications $(i)\Rightarrow (ii)$ hold for arbitrary multi-sequences. If (*ii*) holds and the multi-sequences are isotropic, i.e. $M_\alpha =M_{|\alpha |}$ for any $\alpha \in {\mathbb {N}}_0^d$ and $\textbf{M}\in \mathcal{L}\mathcal{C}$, then the implications $(ii)\Rightarrow (i)$ are valid, too.

#### Remark 4.5

If we consider the *isotropic* setting, i.e. $M^{(\lambda )}_{\alpha }=M^{(\lambda )}_{|\alpha |}$ for any $\lambda >0$ and $\alpha \in \mathbb {N}^d_0$, in all results in this section, then we have that $(ii)\Rightarrow (i)$ is valid if (*ii*) holds for some dimension $d\in \mathbb {N}$. For the analogous results in the anisotropic setting, we refer to [5].

As a consequence, we can deduce the corresponding results for spaces defined by weight functions. We need Theorems 4.1 and 4.3, [4, Lemma 13], Lemmas 2.2 and 2.3 and Proposition 2.4.

#### Corollary 4.6

Let $\omega$ and $\sigma$ be weight functions. Then the following are equivalent:

(i) $$\omega \preceq \sigma $$.
(ii) $\mathcal {S}_{\{\omega \}}(\mathbb {R}^d)\subseteq \mathcal {S}_{\{\sigma \}}(\mathbb {R}^d)$ holds for all dimensions $d\in \mathbb {N}$ with continuous inclusion.
(ii’) $\mathcal {S}_{\{\omega \}}(\mathbb {R})\subseteq \mathcal {S}_{\{\sigma \}}(\mathbb {R})$ holds with continuous inclusion. Under the assumptions $\omega (t)=o(t^2)$, $\sigma =o(t^2)$ as $t\rightarrow \infty$, the previous statements are equivalent to
(iii) $\mathcal {S}_{(\omega )}(\mathbb {R}^d)\subseteq \mathcal {S}_{(\sigma )}(\mathbb {R}^d)$ holds for all dimensions $d\in \mathbb {N}$ with continuous inclusion.
(iii’) $\mathcal {S}_{(\omega )}(\mathbb {R})\subseteq \mathcal {S}_{(\sigma )}(\mathbb {R})$ holds with continuous inclusion.

Now we treat the mixed case between the Roumieu and Beurling classes. By Theorem 4.2, [4, Lemma 13], Lemmas 2.2 and 2.3 and Proposition 2.4, we obtain

#### Corollary 4.7

Let $\omega$ and $\sigma$ be weight functions with $\sigma (t)=o(t^2)$ as $t\rightarrow \infty$. Then the following are equivalent:

(i) $$\omega \vartriangleleft \sigma $$, i.e. $$\sigma (t)=o(\omega (t))$$ as $$t\rightarrow \infty $$,
(ii) $\mathcal {S}_{\{\omega \}}(\mathbb {R}^d)\subseteq \mathcal {S}_{(\sigma )}(\mathbb {R}^d)$ holds for all dimensions $d\in \mathbb {N}$ with continuous inclusion.
(ii’) $\mathcal {S}_{\{\omega \}}(\mathbb {R})\subseteq \mathcal {S}_{(\sigma )}(\mathbb {R})$ holds with continuous inclusion.

## Comparison of Classes Defined by Weight Sequences and Weight Functions

Gathering the information from the previous section, we are now able to prove the following results which are analogous to the statements obtained in [6] and [18] for the spaces $\mathcal {E}_{\{\textbf{M}\}}, \mathcal {E}_{(\textbf{M})}, \mathcal {E}_{\{\omega \}}, \mathcal {E}_{(\omega )}$ (cf. also [4, Remark 4]).

### Theorem 5.1

Let $\omega$ be a weight function with associated weight matrix $\mathcal {M}_{\omega }:=\{\textbf{W}^{(\lambda )}: \lambda >0\}$. Then the following are equivalent:

(i) $$\omega $$ satisfies (3.6),
(ii) There exists an isotropic multi-sequence $\textbf{M}\in \mathcal{L}\mathcal{C}$ such that:
(ii.1) $\textbf{M}$ satisfies (2.4), and hence (2.3);
(ii.2) $$\textbf{M}$$ satisfies (2.8);
(ii.3) $$\omega _{\textbf{M}}$$ satisfies $$(\alpha )$$;
(ii.4) for any $d\in \mathbb {N}$, we have $\mathcal {S}_{\{\omega \}}(\mathbb {R}^d)=\mathcal {S}_{\{\textbf{M}\}}(\mathbb {R}^d)$ as topological vector spaces.

The analogous result holds true for the Beurling case as well, when considering (in addition) $\omega (t)=o(t^2)$ as $t\rightarrow \infty$ for the weight function $\omega$ and condition (2.12) instead of (2.8).

In both cases, we can take $\textbf{M}\equiv \textbf{W}^{(\lambda )}$ for some/each $\lambda >0$ in (*ii*).

### Proof

We will only treat the Roumieu case explicitly. The Beurling case follows analogously.

*The Roumieu case* $(i)\Rightarrow (ii)$: First, by [18, Lemma 5.9 (5.11)], we get that $\mathcal {M}_{\omega }$ is constant, more precisely $\mathcal {M}_{\omega }\{\approx \}\textbf{W}^{(\lambda )}$ for some/each $\lambda >0$. Thus, by definition of the spaces and Proposition 2.4, we get as topological vector spaces for all $d\in \mathbb {N}$

$$\begin{aligned} \mathcal {S}_{\{\omega \}}(\mathbb {R}^d)=\mathcal {S}_{\{\mathcal {M}_{\omega }\}}(\mathbb {R}^d)=\mathcal {S}_{\{\mathbf {W^{(\lambda )}}\}}(\mathbb {R}^d),\qquad \forall \;\lambda >0. \end{aligned}$$

Condition $\textbf{W}^{(\lambda )}\in {\mathcal{L}\mathcal{C}}$ is clear by definition. Moreover, [18, Corollary 5.8 (2)] yields that some/each $\textbf{W}^{(\lambda )}$ satisfies (2.4), hence (2.3) as well. Also (2.8) for some/each $\textbf{W}^{(\lambda )}$ follows by [4, Lemma 13(a)] applied to $r=1/2$ which can be done by assumption $(\beta )$ on $\omega$ and by [4, Proposition 3 $(a)\Rightarrow (b)$].

Finally, that $\omega _{\textbf{W}^{(\lambda )}}$ satisfies $(\alpha )$ for some/each $\lambda >0$ follows by the fact that $(\alpha )$ holds for $\omega$ by assumption, by [18, Lemma 5.7] and because this condition is clearly stable under equivalence of weight functions.

$(ii)\Rightarrow (i)$: First, we want to show that the matrix $\mathcal {M}_{\omega }$ is constant, i.e. $\textbf{W}^{(\lambda )}\approx \textbf{W}^{(\kappa )}$ for all $\lambda ,\kappa >0$.

By Proposition 2.4 and assumption (*ii*.4), we get as topological vector spaces,

$$\begin{aligned} \mathcal {S}_{\{\mathcal {M}_{\omega }\}}(\mathbb {R}^d)=\mathcal {S}_{\{\omega \}}(\mathbb {R}^d)=\mathcal {S}_{\{\textbf{M}\}}(\mathbb {R}^d). \end{aligned}$$

Now Theorem 4.1 applied to the inclusion $\mathcal {S}_{\{\mathcal {M}_{\omega }\}}(\mathbb {R})\subseteq \mathcal {S}_{\{\textbf{M}\}}(\mathbb {R})$ and to $\mathcal {M}\equiv \mathcal {M}_{\omega }$, $\mathcal {N}\equiv \{\textbf{M}\}$, yields $\mathcal {M}_{\omega }\{\preceq \}\textbf{M}$. By the converse inclusion $\mathcal {S}_{\{\textbf{M}\}}(\mathbb {R})\subseteq \mathcal {S}_{\{\mathcal {M}_{\omega }\}}(\mathbb {R})$ and Theorem 4.1 applied to $\mathcal {M}\equiv \{\textbf{M}\}$ and $\mathcal {N}\equiv \mathcal {M}_{\omega }$, we get $\textbf{M}\{\preceq \}\mathcal {M}_{\omega }$ as well.

Recall that we can apply this characterizing result since $\omega$ is assumed to be a weight function and because of (*ii*.1) and (*ii*.2) for $\textbf{M}$.

Summarizing, so far we have shown $\mathcal {M}_{\omega }\{\approx \}\textbf{M}$ which clearly implies that $\mathcal {M}_{\omega }$ is constant. Then [18, Lemma 5.9 (5.11)] yields (3.6) for $\omega$ and $\textbf{M}\approx \textbf{W}^{(\lambda )}$ for some/any $\lambda >0$ follows. $\square$

Conversely, in the next result, we start with a weight sequence, however the required arguments for the proof are the same as before.

### Theorem 5.2

Let $\textbf{M}\in {\mathcal{L}\mathcal{C}}$ be given and set $M_{\alpha }:=M_{|\alpha |}$ for any $\alpha \in \mathbb {N}^d_0$. Assume that:

- $$\textbf{M}$$ satisfies (2.8);
- $$\textbf{M}$$ satisfies (2.3);
- $$\omega _{\textbf{M}}$$ satisfies $$(\alpha )$$.

Then the following are equivalent:

(i) $$\textbf{M}$$ satisfies (2.4);
(ii) there exists a weight function $\omega$ satisfying (3.6) such that for all $d\in \mathbb {N}$
  5.1 $$\begin{aligned} \mathcal {S}_{\{\omega \}}(\mathbb {R}^d)=\mathcal {S}_{\{\textbf{M}\}}(\mathbb {R}^d) \end{aligned}$$
  as topological vector spaces.

The analogous result holds true for the Beurling case as well when $\textbf{M}$ satisfies (2.12) (instead of (2.8)) and (in addition) $\omega (t)=o(t^2)$ as $t\rightarrow \infty$.

In both cases, we can take the weight function $\omega \equiv \omega _{\textbf{M}}$ in (*ii*).

### Proof

Again we only treat the Roumieu case.

$(i)\Rightarrow (ii)$ We consider the weight function $\omega _{\textbf{M}}$. The basic assumptions to be a weight function, $(\gamma )$ and $(\delta )$ hold automatically by definition and $(\alpha )$ follows by assumption (*c*); (2.4) implies (3.6), see [14, Proposition 3.6]. The choice $\beta =0$ in (2.8) for $\textbf{M}$ and the proof of (6.6) in [4, Lemma 13 (a)] applied to $r=1/2$ and to $\textbf{W}^{(\lambda )}\equiv \textbf{M}$ imply $(\beta )$ for $\omega _{\textbf{M}}$.

Thus, $\omega _{\textbf{M}}$ is a weight function as required for (*ii*). Let $\mathcal {M}_{\omega _{\textbf{M}}}:=\{\textbf{M}^{(\lambda )}: \lambda >0\}$ be the matrix associated to $\omega _{\textbf{M}}$. By [18, Lemma 5.9 (5.11)] we get that this matrix is constant and since $\textbf{M}\equiv \textbf{M}^{(1)}$ (see (*V*) in Sect. 3), we have $\textbf{M}^{(\lambda )}\approx \textbf{M}$ for all $\lambda >0$. This shows (5.1) for $\omega \equiv \omega _{\textbf{M}}$ by taking into account [4, Proposition 5].

$(ii)\Rightarrow (i)$ We follow the proof of $(ii)\Rightarrow (i)$ in Theorem 5.1 by applying Theorem 4.1 twice which can be done by the assumptions on $\omega$ and $\textbf{M}$. By (3.6) the associated matrix $\mathcal {M}_{\omega }$ is constant (see [18, Lemma 5.9 (5.11)]), some/each $\textbf{W}^{(\lambda )}$ satisfies (2.4) (see [14, Proposition 3.6]) and finally $\textbf{W}^{(\lambda )}\approx \textbf{M}$ for some/each $\lambda >0$ holds. Since (2.4) is obviously stable under the equivalence relation $\approx$, the proof is complete. $\square$

### Remark 5.3

Recently, in [23, Theorem 3.1], the requirement that $\omega _{\textbf{M}}$ has $(\alpha )$, arising in (*ii*.3) in Theorem 5.1 and in assumption (*c*) in Theorem 5.2, has been characterized in terms of $\textbf{M}\in {\mathcal{L}\mathcal{C}}$ by the following condition:

$$\begin{aligned} \exists \;L\in \mathbb {N}:\;\;\;\liminf _{p\rightarrow \infty }\frac{(M_{Lp})^{1/(Lp)}}{(M_p)^{1/p}}>1. \end{aligned}$$

## Characterization of the Inclusion Relations in the Non-quasianalytic Case

In this section, we present alternative proofs for the characterizations of the inclusion relations for Gelfand–Shilov classes. Here, we are not using results from [4] but following ideas generally used in the ultradifferentiable setting. Our assumptions are slightly different from Sect. 4. In fact, here we need *non-quasianalyticity*, i.e. the existence of non-trivial compactly supported ultradifferentiable functions. This was not required in Sect. 4.

Moreover, although we needed the mixed $(M2)'$ conditions (2.10) and (2.14) in Theorems 4.1 and 4.3, here we can avoid these assumptions. Finally, here the Roumieu and the Beurling cases require different techniques.

### The Roumieu Case

Let $\textbf{M}\in {\mathcal{L}\mathcal{C}}$ be given. We recall (e.g. see [18, Lemma 2.9]): There exists $\theta _{\textbf{M}}\in \mathcal {E}_{\{\textbf{M}\}}(\mathbb {R})$ such that

$$\begin{aligned} \exists \;C,h>0\;\forall \;j\in \mathbb {N}_0\;\forall \;x\in \mathbb {R}:\;\;\;|\theta _{\textbf{M}}^{(j)}(x)|\le Ch^jM_j, \end{aligned}$$

and with $|\theta _{\textbf{M}}^{(j)}(0)|\ge M_j$ for all $j\in \mathbb {N}_0$. In [18], such a function has been called a *characteristic function*. We can assume $\theta _{\textbf{M}}$ to be real- or complex-valued (see the proof of [18, Lemma 2.9]) and note that $\theta _{\textbf{M}}$ cannot belong to the Beurling-type class $\mathcal {E}_{(\textbf{M})}(\mathbb {R})$. Such functions are useful to characterize the inclusion relations of (global/local) ultradifferentiable function classes in terms of growth relations of weight sequences/functions or even matrices, see [18, Propositions 2.12 and 4.6, Corollary 5.17].

However, for our purposes, we need that $\theta _{\textbf{M}}\in \mathcal {S}_{\{\textbf{M}\}}(\mathbb {R})$, $\textbf{M}\in {\mathcal{L}\mathcal{C}}$. To this aim, we assume that $\textbf{M}$ is *non-quasianalytic*, i.e.

6.1 $$\begin{aligned} \sum _{k\ge 1}\frac{1}{\mu _k}=\sum _{k\ge 1}\frac{M_{k-1}}{M_k}<+\infty . \end{aligned}$$

Accordingly, a standard log-convex matrix $\mathcal {M}$ is called *Roumieu non-quasianalytic,* if there exists some $\lambda _0>0$ such that $\textbf{M}^{(\lambda _0)}$ is non-quasianalytic.

By the well-known *Denjoy–Carleman theorem*, we obtain that both the classes $\mathcal {D}_{\{\textbf{M}\}}$ and $\mathcal {D}_{(\textbf{M})}$ are non-trivial, see e.g. [14, Theorem 4.2], and define

6.2 $$\begin{aligned} \psi _{\textbf{M}}:=\theta _{\textbf{M}}\cdot \phi , \end{aligned}$$

with $\phi \in \mathcal {D}_{\{\textbf{M}\}}$ having $\phi ^{(j)}(0)=\delta _{j,0}$ (Kronecker delta). For the existence of such a specific test function, we refer to the proof of [17, Theorem 2.2]. Concerning the support of $\phi$ we do not make any restriction and by involving the product rule (cf. [20, Lemma 2.0.6]) the following statement is then immediate:

#### Lemma 6.1

Let $\textbf{M}\in {\mathcal{L}\mathcal{C}}$ be non-quasianalytic and $\psi _{\textbf{M}}$ the function defined via (6.2). Then $\psi _{\textbf{M}}\in \mathcal {D}_{\{\textbf{M}\}}\subseteq \mathcal {S}_{\{\textbf{M}\}}$ and $|\psi ^{(j)}_{\textbf{M}}(0)|\ge M_j$ for all $j\in \mathbb {N}_0$.

With this preparation, we are able to prove the first main statement.

#### Theorem 6.2

Let $\mathcal {M}:=\{\textbf{M}^{(\lambda )}: \lambda >0\}$, $\mathcal {N}:=\{\textbf{N}^{(\lambda )}: \lambda >0\}$ be arbitrary and consider the following assertions:

(i) $$\mathcal {M}\{\preceq \}\mathcal {N}$$,
(ii) $\mathcal {S}_{\{\mathcal {M}\}}(\mathbb {R}^d)\subseteq \mathcal {S}_{\{\mathcal {N}\}}(\mathbb {R}^d)$ is valid with continuous inclusion.

Then $(i)\Rightarrow (ii)$ is valid for all dimensions $d\in \mathbb {N}$. If $\mathcal {M}$ is standard log-convex and Roumieu non-quasianalytic and if (*ii*) holds for the case $d=1$ (and hence also $\mathcal {N}$ is Roumieu non-quasianalytic), then $(ii)\Rightarrow (i)$ is valid, too.

#### Proof

$(i)\Rightarrow (ii)$ follows again by the definition of the spaces.

For $(ii)\Rightarrow (i)$, we use this inclusion for $d=1$. Since $\mathcal {M}$ is standard log-convex, for each given index $\lambda >0$, we can find $\theta _{\textbf{M}^{(\lambda )}}$. Since $\mathcal {M}$ is Roumieu non-quasianalytic, we can assume that each $\textbf{M}^{(\lambda )}$ is non-quasianalytic as well and so $\mathcal {D}_{\{\textbf{M}^{(\lambda )}\}}$ is non-trivial. This can be achieved by “not considering” all possible quasianalytic sequences $M^{(\lambda )}$, $\lambda <\lambda _0$, which by definition does not change the according function classes. By Lemma, 6.1

$$\begin{aligned} \psi _{\textbf{M}^{(\lambda )}}\in \mathcal {S}_{\{\textbf{M}^{(\lambda )}\}}(\mathbb {R})\subseteq \mathcal {S}_{\{\mathcal {M}\}}(\mathbb {R})\subseteq \mathcal {S}_{\{\mathcal {N}\}}(\mathbb {R}), \end{aligned}$$

hence there exist some $\kappa >0$ and $C,h>0$ such that $|\psi _{\textbf{M}^{(\lambda )}}^{(j)}(x)|\le Ch^jN^{(\kappa )}_j$ for all $x\in \mathbb {R}$ and $j\in \mathbb {N}_0$. For $x=0$, we get $M^{(\lambda )}_j\le |\psi _{\textbf{M}^{(\lambda )}}^{(j)}(0)|$ for all $j\in \mathbb {N}_0$ and both estimates imply $\textbf{M}^{(\lambda )}\preceq \textbf{N}^{(\kappa )}$. $\square$

Let $\omega$ be a (general) weight function, we call $\omega$ *non-quasianalytic*, if

6.3 $$\begin{aligned} \int _{1}^{\infty }\frac{\omega (t)}{t^2}dt<+\infty . \end{aligned}$$

It is known, see e.g. [21, Corollary 4.8], that $\omega$ is non-quasianalytic if and only if some/each $\textbf{W}^{(\lambda )}$ is non-quasianalytic; i.e. if and only if $\mathcal {M}_{\omega }$ is Roumieu non-quasianalytic. Since $\mathcal {M}_{\omega }$ is always standard log-convex (see Lemma 2.3), Theorem 6.2 implies

#### Corollary 6.3

Let $\omega$ and $\sigma$ be non-quasianalytic weight functions. Then the following are equivalent:

(i) $$\omega \preceq \sigma $$,
(ii) $\mathcal {S}_{\{\omega \}}(\mathbb {R}^d)\subseteq \mathcal {S}_{\{\sigma \}}(\mathbb {R}^d)$ is valid for all $d\in \mathbb {N}$ with continuous inclusion.

$(i)\Rightarrow (ii)$ is valid for general weight functions $\omega$ and $\sigma$ and for $(ii)\Rightarrow (i)$ only $d=1$ is required.

### The Beurling Case

We call a standard log-convex weight matrix $\mathcal {M}=\{\textbf{M}^{(\lambda )}: \lambda >0\}$ *Beurling non-quasianalytic,* when for all $\lambda >0$ the sequence $\textbf{M}^{(\lambda )}$ is non-quasianalytic. This definition is justified by [21, Theorem 4.1, Sect. 4.6]: A countable intersection of non-quasianalytic ultradifferentiable classes (with totally ordered weight sequences) is still non-quasianalytic. So, if $\mathcal {M}$ is standard log-convex and *Beurling non-quasianalytic,* then

$$\begin{aligned} \mathcal {D}_{(\mathcal {M})}:=\mathcal {D}\cap \mathcal {E}_{(\mathcal {M})}=\mathcal {D}\cap \bigcap _{\lambda >0}\mathcal {E}_{(\textbf{M}^{(\lambda )})}=\mathcal {D}\cap \bigcap _{n\in \mathbb {N}}\mathcal {E}_{(\textbf{M}^{(1/n)})} \end{aligned}$$

is non-trivial; see [21, Propositions 4.7 (i) and  4.4]. (Recall that for huge intersections this statement fails in general.) For any given weight function $\omega$, the associated matrix $\mathcal {M}_{\omega }$ is Beurling non-quasianalytic if and only if $\omega$ is non-quasianalytic.

Let us now consider the following Beurling-type condition:

6.4 $$\begin{aligned} \forall \;\lambda>0\;\exists \;\kappa >0\;\exists \;A\ge 1\;\forall \;p\in \mathbb {N}_0:\;\;\;(M^{(\kappa )}_p)^2\le A^pM^{(\lambda )}_p. \end{aligned}$$

It is immediate to see that $\mathcal {M}=\{\textbf{M}\}$, $\textbf{M}\in {\mathcal{L}\mathcal{C}}$, can never satisfy (6.4) because this would yield $\sup _{p\ge 1}(M_p)^{1/p}<+\infty$.

Moreover, if $\mathcal {M}$ is standard log-convex and satisfies (2.12), then for all $\lambda >0$ there exist $H>0$ and $B>0$ such that $j^{j/2}\le BH^{j}M^{(\lambda )}_{j}$ for all $j\in \mathbb {N}_0$, i.e. $\textbf{G}^{1/2}\preceq \textbf{M}^{(\lambda )}$. Thus, it is immediate to see that any standard log-convex matrix having (6.4) and (2.12) is Beurling non-quasianalytic.

Now we are ready to state the following result which is analogous to Theorem 6.2.

#### Theorem 6.4

Let $\mathcal {M}$ and $\mathcal {N}$ be arbitrary and consider the following assertions:

(i) $$\mathcal {M}(\preceq )\mathcal {N}$$,
(ii) $\mathcal {S}_{(\mathcal {M})}(\mathbb {R}^d)\subseteq \mathcal {S}_{(\mathcal {N})}(\mathbb {R}^d)$ is valid with continuous inclusion.

Then $(i)\Rightarrow (ii)$ holds. If both matrices are standard log-convex, $\mathcal {M}$ is Beurling non-quasianalytic and satisfies (6.4) and if (*ii*) holds for the case $d=1$ (and hence also $\mathcal {N}$ is Beurling non-quasianalytic), then $(ii)\Rightarrow (i)$ is valid too.

#### Proof

$(i)\Rightarrow (ii)$ is again clear by the definition of the spaces. Concerning $(ii)\Rightarrow (i)$, we follow the ideas of the proof given in [18, Proposition 4.6 (2)] which is based on techniques developed in [8, Sect. 2]. By the continuous inclusion $\mathcal {S}_{(\mathcal {M})}(\mathbb {R})\subseteq \mathcal {S}_{(\mathcal {N})}(\mathbb {R})$, we get the following:

6.5 $$\begin{aligned}{} & {} \forall \;\lambda>0\;\forall \;h>0\;\exists \;\kappa>0\;\exists \;C,\;h_1>0\;\forall \;f\in \mathcal {S}_{(\mathcal {M})}(\mathbb {R}):\nonumber \\ \quad{} & {} \Vert f\Vert _{\infty ,\textbf{N}^{(\lambda )},h}\le C\Vert f\Vert _{\infty ,\textbf{M}^{(\kappa )},h_1}. \end{aligned}$$

We will apply (6.5) for $h=1$ and to the following family of functions. For each $a>0$, arbitrary but from now on fixed, we consider a function $\phi _a\in \mathcal {D}_{(\mathcal {M})}$ with ${\text {supp}}(\phi _a)\subseteq [-a,a]$ and $\phi _a^{(j)}(0)=\delta _{j,0}$: The existence of such functions follows again by [17, Theorem 2.2], more precisely let $\phi _a\in \mathcal {D}_{\{\textbf{L}\}}\subseteq \mathcal {D}_{(\mathcal {M})}$ with $\textbf{L}\in {\mathcal{L}\mathcal{C}}$, $\textbf{L}\vartriangleleft \mathcal {M}$ denoting the non-quasianalytic sequence constructed in [21, Propositions 4.7 (i) and 4.4]. (Here we use the fact that $\mathcal {M}$ is Beurling non-quasianalytic.) Moreover, for $t\ge 0$ and $x\in \mathbb {R}$, we set $f_t(x):=\exp (itx)$ and finally

$$\begin{aligned} g_{a,t}(x):=f_t(x)\cdot \phi _a(x). \end{aligned}$$

First, it is easy to see $f_t\in \mathcal {E}_{(\mathcal {M})}(\mathbb {R})$ and ${\text {supp}}(g_{a,t})\subseteq [-a,a]$ for all $t\ge 0$. We fix $t\ge 0$ and $a>0$ and by the product rule it is known and straight-forward to verify $g_{a,t}\in \mathcal {D}_{(\mathcal {M})}\subseteq \mathcal {S}_{(\mathcal {M})}$. We apply (6.5) to this family with $h=1$ and set $a:=h_1(\le 1)$.

According to the index $\kappa$ arising in (6.5), by applying (6.4), we get an index $\kappa _1$ and $A\ge 1$ such that $(M^{(\kappa _1)}_p)^2\le A^pM^{(\kappa )}_p$ for any $p\in \mathbb {N}_0$.

Using this preparation, we start now by estimating the right-hand side in (6.5) for all $t\ge 1$:

6.6 $$\begin{aligned} C\Vert g_{h_1,t}\Vert _{\infty ,\textbf{M}^{(\kappa )},h_1}= & {} C\sup _{j,k\in \mathbb {N}_0}\sup _{x\in \mathbb {R}} \frac{|x^jg_{h_1,t}^{(k)}(x)|}{h_1^{j+k}M^{(\kappa )}_{j+k}}=C\sup _{j,k\in \mathbb {N}_0} \sup _{x\in [-h_1,h_1]}\frac{|x^jg_{h_1,t}^{(k)}(x)|}{h_1^{j+k}M^{(\kappa )}_{j+k}}\nonumber \\\le & {} C\sup _{j,k\in \mathbb {N}_0}\sup _{x\in [-h_1,h_1]}\frac{h_1^j|g_{h_1,t}^{(k)}(x)|}{h_1^{j+k}M^{(\kappa )}_{j+k}} =C\sup _{j,k\in \mathbb {N}_0}\sup _{x\in [-h_1,h_1]}\frac{|g_{h_1,t}^{(k)}(x)|}{h_1^{k}M^{(\kappa )}_{j+k}}\nonumber \\\le & {} CC_{h_1,\kappa _1}\sup _{j,k\in \mathbb {N}_0}\frac{t^k(1+h_1)^kM^{(\kappa _1)}_k}{h_1^kM^{(\kappa )}_{j+k}} \le CC_{h_1,\kappa _1}\sup _{k\in \mathbb {N}_0}\frac{(2t)^kM^{(\kappa _1)}_k}{h_1^kM^{(\kappa )}_k}\nonumber \\\le & {} CC_{h_1,\kappa _1}\sup _{k\in \mathbb {N}_0}\frac{(2tA)^k}{h_1^kM^{(\kappa _1)}_k}=CC_{h_1,\kappa _1} \exp \left( \omega _{\textbf{M}^{(\kappa _1)}}(2At/h_1)\right) .\nonumber \\ \end{aligned}$$

To estimate the second inequality of (6.6), we argued as follows: Since $\phi _{h_1}\in \mathcal {D}_{(\mathcal {M})}\subseteq \mathcal {S}_{(\mathcal {M})}(\mathbb {R})$, we get

$$\begin{aligned} |g_{h_1,t}^{(k)}(x)|\le & {} \sum _{l=0}^k\left( {\begin{array}{c}k\\ l\end{array}}\right) t^l|\phi _{h_1}^{(k-l)}(x)|\le \sum _{l=0}^k \left( {\begin{array}{c}k\\ l\end{array}}\right) t^lC_{h_1,\kappa _1}h_1^{k-l}M^{(\kappa _1)}_{k-l}\\\le & {} C_{h_1,\kappa _1}M^{(\kappa _1)}_kt^k\sum _{l=0}^k\left( {\begin{array}{c}k\\ l\end{array}}\right) h_1^{k-l}=C_{h_1,\kappa _1}t^kM^{(\kappa _1)}_k(1+h_1)^k. \end{aligned}$$

Note that by log-convexity and normalization $M^{(\kappa _1)}_{k-l}\le M^{(\kappa _1)}_k$, i.e. each sequence is increasing and since we are dealing with the Beurling case we will have $0<h_1\le 1$ (small). Moreover, note that we have estimated by $\frac{1}{M^{(\kappa )}_{j+k}}\le \frac{1}{M^{(\kappa )}_{k}}$ for any $j,k\in \mathbb {N}_0$ and any index $\kappa$.

We continue now with the left-hand side in (6.5) and get

$$\begin{aligned} \Vert g_{h_1,t}\Vert _{\infty ,\textbf{N}^{(\lambda )},1}&=\sup _{j,k\in \mathbb {N}_0}\sup _{x\in \mathbb {R}}\frac{|x^jg_{h_1,t}^{(k)}(x)|}{N^{(\lambda )}_{j+k}}\\ {}&\underbrace{\ge }_{x=0=j}\sup _{k\in \mathbb {N}_0}\frac{|g_{h_1,t}^{(k)}(0)|}{N^{(\lambda )}_{k}}=\sup _{k\in \mathbb {N}_0}\frac{t^k}{N^{(\lambda )}_{k}}=\exp \left( \omega _{\textbf{N}^{(\lambda )}}(t)\right) . \end{aligned}$$

Summarizing, we have shown that (6.5) yields:

$$\begin{aligned}&{} \forall \;\lambda>0\;\exists \;\kappa _1>0\;\exists \;C,A,h_1>0\;\forall \;t\ge 1:\\{}&{} \qquad \qquad \qquad \exp \left( \omega _{{\textbf {N}}^{(\lambda )}}(t)\right) \le C\exp \left( \omega _{{\textbf {M}}^{(\kappa _1)}}(2At/h_1)\right) . \end{aligned}$$

Since $\textbf{N}^{(\lambda )}\in {\mathcal{L}\mathcal{C}}$ (for any $\lambda >0$) we get $\omega _{\textbf{N}^{(\lambda )}}(t)=0$ for all $t\in [0,1]$. So the above estimate is valid for all $t\ge 0$. Finally, we are applying (2.6) and obtain for any $p\in \mathbb {N}_0$ that

$$\begin{aligned} N^{(\lambda )}_p= & {} \sup _{t\ge 0}\frac{t^p}{\exp (\omega _{\textbf{N}^{(\lambda )}}(t))}\ge \frac{1}{C}\sup _{t\ge 0}\frac{t^p}{\exp (\omega _{\textbf{M}^{(\kappa _1)}}(2At/h_1))}\\= & {} \frac{1}{C}\left( \frac{h_1}{2A}\right) ^p\sup _{s\ge 0}\frac{s^p}{\exp (\omega _{\textbf{M}^{(\kappa _1)}}(s))} =\frac{1}{C}\left( \frac{h_1}{2A}\right) ^pM^{(\kappa _1)}_p, \end{aligned}$$

which implies $\mathcal {M}(\preceq )\mathcal {N}$ and finishes the proof. $\square$

Again, by involving the associated weight matrices, we can transfer Theorem 6.4 to the weight function situation:

#### Corollary 6.5

Let $\omega$ be a weight function with

6.7 $$\begin{aligned} \exists \;H>0:\;\;\;\omega (t^2)=O(\omega (Ht)),\;\;\;t\rightarrow +\infty , \end{aligned}$$

and $\sigma$ be a (non-quasianalytic) weight function. Then the following are equivalent:

(i) $$\omega \preceq \sigma $$,
(ii) $\mathcal {S}_{(\omega )}(\mathbb {R}^d)\subseteq \mathcal {S}_{(\sigma )}(\mathbb {R}^d)$ holds for all dimensions $d\in \mathbb {N}$ with continuous inclusion.

$(i)\Rightarrow (ii)$ is valid for general weight functions $\omega$ and $\sigma$. For $(ii)\Rightarrow (i)$, only the inclusion for $d=1$ is required.

#### Proof

In order to apply Theorem 6.4 to the matrices $\mathcal {M}_{\omega }$ and $\mathcal {M}_{\sigma }$, we remark that, by [12, Appendix A], $\omega$ satisfies (6.7) if and only if $\mathcal {M}_{\omega }$ satisfies (6.4) and $\omega$ satisfies (6.3), see [12, Lemma A.1]. $\square$

#### Remark 6.6

By [12, Lemma A.1], condition (6.7) is stronger than non-quasianaliticity, which in turn is stronger than $(\beta )$.

We observe that in Theorems 4.1, 4.2, 4.3 and 6.4 we could avoid the assumption that $\mathcal {N}$ is standard log-convex by substituting (2.6) with

6.8 $$\begin{aligned} N_p\ge \sup _{t\ge 0}\frac{t^p}{\exp (\omega _{\textbf{N}}(t))},\;\;\;p\in \mathbb {N}_0, \end{aligned}$$

which is always true (cf. [5]).

Finally, it is known that $\mathcal {S}_{(\omega )}=\mathcal {S}$, the classical Schwartz class, when the weight $\omega (t)=\log (1+t)$, $t\ge 0$. This *weight* clearly satisfies the standard assumptions $(\alpha )$ and $(\delta )$ in Definition 2.1, and is non-quasianalytic, which implies $(\beta )$, but does not satisfy $(\gamma )$, nor property (3.6). In fact, $\mathcal {M}_{\omega }$ is not a weight matrix as defined in Section 2.3, because the matrix associated with this weight does not contain sequences of positive numbers. Moreover, due to [22, Lemma 7.2], there is no sequence $\textbf{M}\in {\mathcal{L}\mathcal{C}}$ such that

6.9 $$\begin{aligned} \omega _{\textbf{M}}\sim t\mapsto \log (1+t). \end{aligned}$$

However, if $\textbf{M}=(M_p)_p$ is a sequence with $1=M_0$ and

$$\begin{aligned} \exists \;q_0\in \mathbb {N}_{>0}\;\forall \;p>q_0:\;\;\;M_p=+\infty , \end{aligned}$$

and such that $1\le \mu _p\le \mu _{p+1}$ for $1\le p\le q_0$, then $\omega _{\textbf{M}}$ satisfies (6.9) with the conventions $\frac{1}{+\infty }=0$ and $\log (0)=-\infty$. Hence, [4, Lemma 12] remains valid, and so $\omega _{\textbf{W}^{(\lambda )}}\sim \omega$ for all indices $\lambda >0$.

## Data Availability

The manuscript has no associated data.
